# Fast yet force-effective mode of supracellular collective cell migration due to extracellular force transmission

**DOI:** 10.1371/journal.pcbi.1012664

**Published:** 2025-01-09

**Authors:** Amrit Bagchi, Bapi Sarker, Jialiang Zhang, Marcus Foston, Amit Pathak

**Affiliations:** 1 Department of Mechanical Engineering & Materials Science, Washington University, St. Louis, Missouri, United States of America; 2 Department of Energy, Environmental & Chemical Engineering, Washington University, St. Louis, Missouri, United States of America; Oxford, UNITED KINGDOM OF GREAT BRITAIN AND NORTHERN IRELAND

## Abstract

Cell collectives, like other motile entities, generate and use forces to move forward. Here, we ask whether environmental configurations alter this proportional force-speed relationship, since aligned extracellular matrix fibers are known to cause directed migration. We show that aligned fibers serve as active conduits for spatial propagation of cellular mechanotransduction through matrix exoskeleton, leading to efficient directed collective cell migration. Epithelial (MCF10A) cell clusters adhered to *soft* substrates with aligned collagen fibers (AF) migrate faster with much lesser traction forces, compared to random fibers (RF). Fiber alignment causes higher motility waves and transmission of normal stresses deeper into cell monolayer while minimizing shear stresses and increased cell-division based fluidization. By contrast, fiber randomization induces cellular jamming due to breakage in motility waves, disrupted transmission of normal stresses, and heightened shear driven flow. Using a novel motor-clutch model, we explain that such ‘force-effective’ fast migration phenotype occurs due to rapid stabilization of contractile forces at the migrating front, enabled by higher frictional forces arising from simultaneous compressive loading of parallel fiber-substrate connections. We also model ‘*haptotaxis*’ to show that increasing ligand connectivity (but not continuity) increases migration efficiency. According to our model, increased rate of front stabilization via higher resistance to substrate deformation is sufficient to capture ‘*durotaxis’*. Thus, our findings reveal a new paradigm wherein the rate of leading-edge stabilization determines the efficiency of supracellular collective cell migration.

## Author summary

Cellular forces are critical for their collective migration on matrices of various stiffness and topographies. While migrating cells can align collagen fibers, it remains unknown whether such force requirement changes with pre-aligned fibers. On soft surfaces with aligned collagen fibers, we discovered a new mode of fast collective cell migration that requires drastically lower traction forces, compared to surfaces without aligned fibers. Through traction force and monolayer stress microscopy calculations and a new motor-clutch model, we further discovered that this novel mode of “fast and force-effective” collective cell migration is achieved by rapid stabilization of cellular contractile forces via simultaneous engagement of fiber-substrate linkages when cells pull on aligned fibers. This phenomenon results in higher fluidization and stress relaxation within the monolayer, allowing the monolayer to expand freely. Although high traction forces have conventionally been associated with fast cell migration, our findings show that aligned fibers relax that force requirement via fast contractility stabilization.

## Introduction

Collective cell populations in response to various physical and chemical cues undergo directional migration that drives critical steps in development, repair, and disease [1,2]. Cells within a collective population undergo migration in two broad phenotypes, which can operate concurrently or exclusively. First, in the presence of stable cell-cell junctions, cells behave as coupled systems driven by dynamic interplay between the active forces at the boundaries and the reciprocal coupling forces between neighboring cells [3–6]. Secondly, with weak or absent cell-cell junctions, cells can behave as nematic-like autonomous bodies that move in response to mutual collisions and self-correction of directionality [7–10]. A parameter for distinguishing these different forms of collective migration is the length scale of front-rear polarity [11]. For example, cell systems resembling active nematic would be driven by individual front-rear polarity [7–10]. By contrast, a collective system of coupled cells consists of two distinct populations, one of leader cells of defined front-rear polarity generating active forces and other of passive follower cells lacking polarity [12–14]. Here, the extent of polarity can be defined in terms of the length scale of transmission of normal stresses propagating from the leading edge into the monolayer, which is attributed to physical intercellular communication via cell-cell junctions [15]. In the event of disrupted intercellular transmission, residual stresses arise, and their shear components determine the direction of flow [15], leading to rich jamming-unjamming transitions [16], antiparallel flows [17], vortices [18] and swirls [13] in migrating epithelia. More recently, a new mode of directional migration has been demonstrated in neural crest cells of *Xenopus* [19]; here, grouped cells show extraordinary supracellular directionality due to continuity in cytoskeletal structures across coupled cells. However, the force-based mechanism behind supracellular migration is yet to be understood. More specifically, since long-range force transmission is crucial for directed collective migration, it remains an important gap in knowledge how force propagation regulates supracellular migration, which is arguably a macroscale mode of directed migration. As such, experimental and modeling frameworks connecting force generation to overall efficiency of collective cell migration remain incomplete.

Extracellular matrix fibers in tissues and organs provide biochemical and structural support to the cellular constituents. Single cells can use these fibers to sense, transmit forces and receive feedback from soft basal matrix stiffness up to 10 μm away from their immediate vicinity to modulate their migration characteristics [20]. Meanwhile, cell collectives can also self-generate aligned fibers from non-aligned structures and use them for long-range force sensing to communicate with other cells (250–1000 μm apart) [21]. As a result, aligned fibers promote long-range directed cell migration observed in cancer metastasis [22,23], wound healing [24,25], branching morphogenesis, and angiogenesis [26,27]. Given the extraordinary length-scales of signal transduction and migrational directionality on aligned fibers, it is likely that cell collectives utilize supracellular modes of migration. According to previous studies, matrix stiffness and chemokine gradient regulate directional migration due to rise in migratory persistence via increased focal adhesion strength [28] and chemotaxis via increased migration-front stability [29,30]. However, it is unknown how aligned topographies of matrix fibers, with constant stiffness or chemokine conditions, regulate directional migration. This is partly because of the inability to accurately quantify cellular migration and forces in 3D, and it remains difficult to devise reductionist soft 2D matrices with surfaces coated with aligned fibers where force measurements are viable.

Using modified polyacrylamide gels capable of attaching aligned fibers, we show that cell clusters apply lesser forces yet migrate faster using a supracellular mode of migration. These results are surprising given the conventionally understood proportional force-speed relationship in cell migration, wherein higher traction forces are often associated with higher metastatic and migratory potential [31]. Moreover, existing literature studying directional collective migration in confinement [18], aligned topographies [32], suggest higher forces are needed for faster migration in narrower confinement or aligned nanogroove topographies. Thus, the mechanism governing collective migration on aligned fibers is distinct from those previously understood, and physics-based explanations of cellular migration dynamics and force generation in combination with novel computational modeling are required to understand the force-effective mode of collective cell migration unveiled in this work. In this mode, the entire monolayer behaves like a single giant polarized unit with cellular contraction occurring up to 500 μm away from the leading edge at the monolayer middle reminiscent of polarized migration of single cells. This migration phenotype is mediated by a global reduction in shear stress and an increase in fluidization. Using a novel motor-clutch model, we show that ligand engagement upon pulling of aligned fibers increases local friction, which in turn reduces traction forces and gives rise to higher cell velocity and overall migration efficiency.

## Results

### Aligned collagen fibers on soft hydrogels enhance collective cell migration speed and persistence

**Fig 1:**
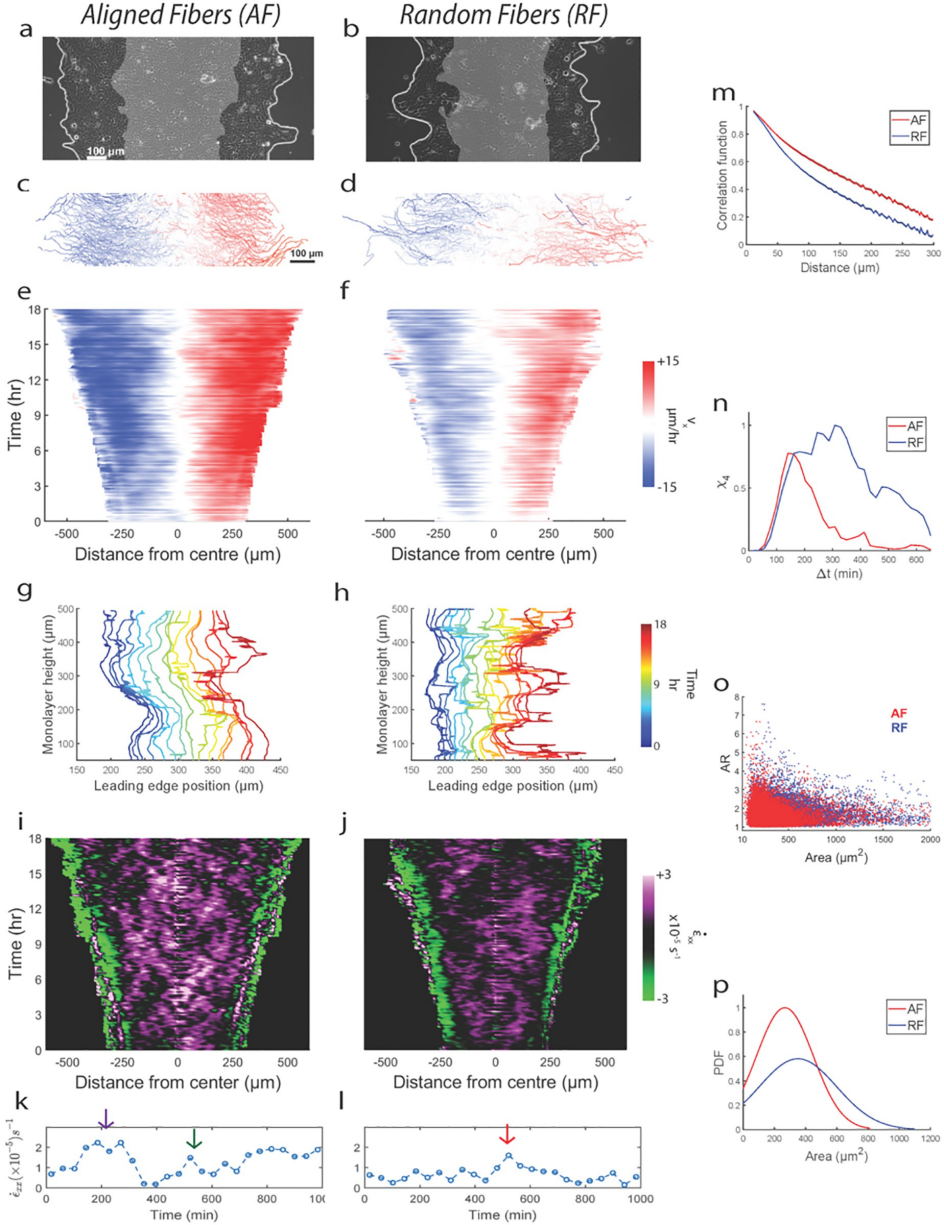
Aligned fibers cause faster and directed cell motion. Representative epithelial monolayer expanding over 16hr (gray region indicates initial position, t = 0 hr) on gels with (**a**) aligned fibers (AF) and (**b**) random fibers (RF). (**c,d**) Corresponding individual cell trajectories, with color-coded mean cell speed in x-direction. Kymographs of cell velocity component in x-direction (*v*_*x*_) for AF (**e**) and RF (**f**).). Plot showing temporal evolution of the leading-edge (average of left and right edges) contour for monolayer migrating on AF (**g**) and RF (**h**) Kymographs for velocity strain rate ${\dot{\varepsilon }}_{x}$ for AF (**i**) and RF (**j**). Plot showing temporal evolution of ${\dot{\varepsilon }}_{xx}$ (averaged across a 100 μm wide strip at the monolayer midline) on AF (**k**) and RF (**l)**. (**m**) Plot comparing time-averaged (m = 157) spatial autocorrelation function of *v*_*x*_ for AF (red) and RF (blue) for n = 3. (**n**) plot comparing four-point susceptibility *χ*_4_ versus Δ*t* between AF and RF. (**o**) Scatter plots of aspect ratio (AR) versus area for cells on AF (m = 56117) and RF (m = 39447). (**p**) Plot comparing cellular area distribution on AF and RF. Refer to statistical methods section for description about m and n.

To probe cellular force propagation through matrix fibers, we aligned collagen-1 fibers to serve as directional cues for cell migration on soft 2D surfaces. We synthesized modified soft polyacrylamide (mod-PA) hydrogels that enable attachment of tunable collagen fibers [33], incubated in 0.1 mg/ml collagen-1, and exposed 11T magnetic field (16 hours) during collagen fibrillogenesis at 4° C (S1a Fig) [34], resulting in aligned long collagen fibers (AF) on soft gels. Using magnetic polydimethyl-siloxane (PDMS) stencils, we micro-patterned rectangular clusters (500 μm wide) of human mammary epithelial cells (MCF-10A) migrating parallel to collagen alignment (S1b, S1c, S1d and S1e Fig). We chose MCF10a cells because aligned fibers are highly relevant in dictating breast cancer metastasis in-vivo [35]. On the other hand, healthy breast tissue has disordered random arrangement of collagen fibers. Thus, for comparison, gels with un-aligned short, random fibers (RF) were used (S1 Fig). While there is an increase in collagen production in metastatic breast tissue [35], here we used same collagen concentration (0.1 mg/ml) across AF and RF. We have not considered changes in local collagen concentration due to fiber alignment and its effect on cell migration. To better understand large-scale spatiotemporal fluctuations of mechanical patterns within the expanding monolayer, we averaged velocities, tractions, and monolayer stresses across length (y-coordinate), thereby reducing dimensionality of system to a single spatial dimension (x-axis, parallel to collagen alignment) and time (Fig 1e, 1f, 1i and 1j).

We found greater cell migration on AF over the entire 18-hour duration of migration (compared to RF; Figs 1a, 1b, S2a and S2b). By comparing cell migration trajectories (Fig 1c and 1d), we observed that cell clusters on AF migrated faster (*1.56 times) compared to RF. Additionally, migration persistence along x-axis was higher on AF (by 23%) (S1c Fig), indicating that cells use aligned fibers as directional cues to guide their migration towards greater collective expansion. Velocity kymographs suggested cell movements for AF were more homogenous with higher velocities at the leading edge penetrating greater depths within the monolayer, compared to higher heterogeneity in RF characterized by outward faster velocities and middle regions of lesser velocities (Fig 1e and 1f). Quantitatively, we measured the spatial velocity autocorrelation function ($C\left(R\right)={\left(Nvar(\bar{v})\right)}^{-1}\sum_{i,j=1}^{N}\left[\sum_{\left|R_{i}-R_{j}\right|}\delta \bar{v_{i}}\delta \bar{v_{j}}\right]$), which follows a slower spatial decay in AF (correlation length higher by 26%), confirming that cellular motion is correlated across greater distances when compared to RF (Fig 1m). Together, velocity kymographs and correlation plots show that cellular motion on AF consists of rapidly propagating cells throughout the depth of the monolayer while on RF, such rapid cells exist at the monolayer edge while the interior consists of slow non-propagating cells. We also studied the temporal evolution of the monolayer front over time for both AF and RF (Fig 1g and 1h). We found greater spatial overlap of the leading front (along the width, x direction) on RF compared to AF where the front maintains its shape (along the height, y direction) across the duration of migration. Thus, the leading front on AF is more temporally stable and smoother in shape compared to RF. Consistent with recent studies connecting migration front stability to a chemotaxis-like directional migration [29,30], we show that aligned fibers also enhance migration front stability and cause faster directed migration than random fibers.

### Collagen fiber alignment enables efficient long-range transmission of unjammed collective cell migration

To investigate temporal heterogeneity in cell movements across monolayer, we use the four-point susceptibility χ_4_, a soft matter physics tool for measuring the rate of structural rearrangements between any two particles (cells) in space within a time window. We computed χ_4_ from the variance of self-overlap order parameter (χ_4_ (Δ*t*) = *N*[〈*Q*(Δ*t*)^2^〉 − 〈*Q*(Δ*t*)〉^2^], where *$Q\left(\Delta t\right)=N^{-1}\sum_{i=1}^{N}w_{i}$*, *w* = 1(0) if |*r*_*i*_ (*t* + Δ*t*)−*r*_*i*_ (*t*)|< 0.9*d_*c*_, *r*_*i*_ (*t*) is x-position of cell at time *t* and *d*_*c*_ is average cell diameter).

While the χ_4_ peak location indicates time of overall structural relaxation, the peak height denotes spatial extent of the rearranging region [36]. Comparing AF to RF, there is a distinct shift in χ_4_ peak to longer timescales for RF (Fig 1n), indicating slower relaxing glassy regime (higher cellular jamming) on RF. Additionally, higher peak height for RF indicates larger clusters of jammed cells. According to previous work on inert particulate matter [37,38], differences in mutual crowding between two conditions could be responsible for distinct jamming states, with denser particle clusters causing higher jamming. Compared to AF, cells on RF had much higher area (31% higher) (Fig 1o and 1p) and slightly higher aspect ratio (2.3% higher) (Figs 1o and S2e) along with much lesser densities (33% lower) (S2d Fig). Although RF cellular morphologies agree with conventional jammed state [39], reduced crowding is counter-intuitive because jamming tends to occur at higher densities in inert systems [38,40]. Based on this experimental evidence, we decided to explore beyond the density-based jamming hypothesis to understand how fiber alignment regulates jamming/unjamming. It is possible that owing to smaller sizes, cells on AF find it easier to slip past each other despite higher densities. In comparison, on RF, relatively larger cell sizes coupled with uncorrelated migration in x- and y-direction can lead to higher impinging among cells, thus biasing the phenotype towards jamming [41]. For cells on AF, our analysis suggests a flocking-like migratory phenotype [42,43], which is spatially homogenous and temporally dynamic. By contrast, on RF, migration is spatially more heterogeneous and static. We will investigate the cause and implications of cell density differences between RF and AF cases later in the manuscript, along with the underlying cause of cellular flocking/jamming on respective fiber alignment conditions.

To further probe the differences in velocity propagation across AF and RF, we investigated the differences in the rate of cell deformation which can be measured from strain-rate (${\dot{\varepsilon }}_{xx}=\partial v_{x}/\partial x$) because of continuity in cell monolayer. Previous studies have revealed the existence of diagonal bands of wavefronts forming X-shaped patterns travelling from monolayer front to back indicating relay in cellular motion via cell-cell junctions [44,45]. Interestingly, for AF alone, there were multiple such strain-rate X-waves [45] propagating away from leading edge, coalescing in the monolayer middle, and then propagating back to edges (Fig 1i and 1j). These X-waves did not have well defined shape for RF condition, indicating breakage in wave propagation. Since X-waves start at the leading edge and propagate to the center, we compared the amount of wave propagation across AF and RF by measuring the strain-rate fluctuations at a 100 μm wide region at the middle of the monolayer (Fig 1k and 1l). We observed two peaks for AF inside first 10 hours of migration compared to a single peak on RF which occurred at the 10^th^ hour time point (arrows in Fig 1k and 1l) indicating faster and more frequent relay of deformation waves on AF. Previously we saw that, monolayer is both faster and denser on AF. Thus, X-waves travel faster, more frequently and across greater cell-cell junctions on AF, which taken together indicate higher efficiency in velocity transmission from front to rear, compared to RF.

Our results indicate that fiber alignment promotes faster migration, greater persistence, and longer transmission of motion within the monolayer while randomizing fiber orientation limits all the above physical parameters. All these differences are further supported by the unjammed/jammed phenotype on AF/RF [46] as well as the differing migration front stability in both conditions. Since migratory fronts are contractile in nature and responsible for force generations in active systems, we wanted to investigate whether the observed differences in migration arise from differences in force generation and their propagations within the monolayer [15].

### Aligned fibers reduce and polarize cellular traction stresses while increasing stress cooperativity

**Fig 2:**
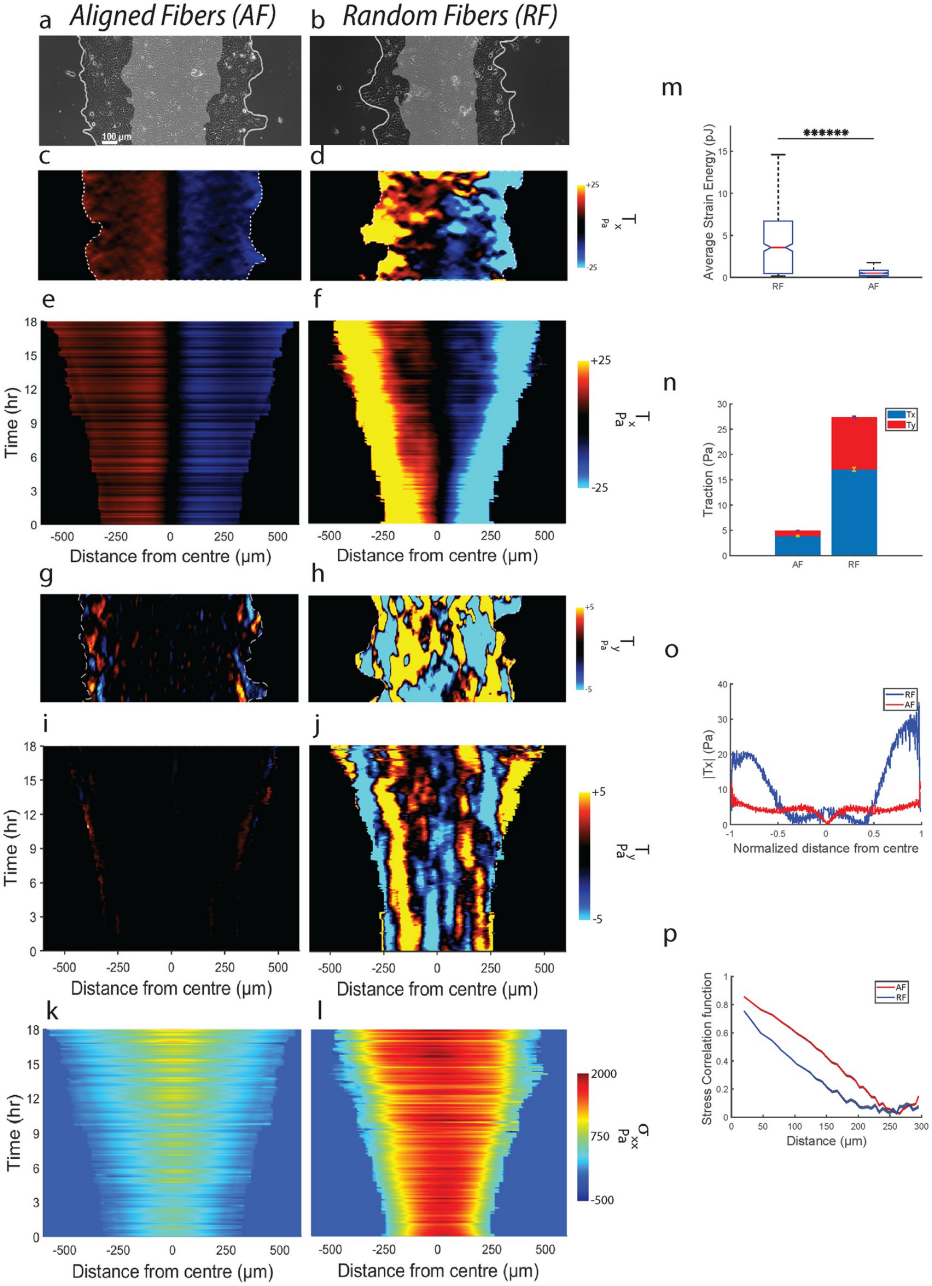
Aligned fibers cause force-effective collective migration. Representative phase contrast images of monolayer expanding on (**a**) AF and (**b**) RF. Representative heatmaps of traction component *T*_*x*_ for (**c**) AF and (**d**) RF. Kymographs for *T*_*x*_ on (**e**) AF and (**f**) RF. Representative heatmaps of traction component *T*_*y*_ for (**g**) AF and (**h**) RF. Kymographs for *T*_*y*_ on (**i**) AF and (**j**) RF. Kymographs for monolayer stress component *σ*_*xx*_ on (**k**) AF and (**l**) RF. (**m**) Plot comparing strain energy between AF and RF (m = 157) across n = 3 (*****P = 1.98x10^-84^). (**n**) Plot comparing *T*_*x*_ with *T*_*y*_ for AF and RF (both *T*_*x*_ and *T*_*y*_ averaged across m = 157 and n = 3). (**o**) Plot comparing time-averaged (m = 157) spatial evolution of *T*_*x*_ along the normalized width of the monolayer (n = 3) between AF and RF. (**p**) Plot comparing time averaged (m = 157) spatial correlation function of average-normal stresses between AF and RF (n = 3). Refer to statistical methods section for description about m and n.

To quantify cellular force generation, we used traction force microscopy [4]. We found that cells on AF exerted significantly lesser traction forces in both x-direction (*T*_*x*_, Fig 2c, 2d, 2e and 2f) and y-direction (*T*_*y*_, Fig 2g, 2h, 2i and 2j). Differences in traction magnitudes are further reflected in total strain energy plot (Fig 2m) showing that cells on AF apply 1/5^th^ of the total strain energy that they applied on RF. Further, comparing *T*_*x*_ and *T*_*y*_ for both conditions, cells on AF apply negligible forces in the y-direction compared to the x-direction (*T*_*y*_ *≈* 0.2*T_*x*_), while cells on RF apply comparable forces in x and y-direction (*T*_*y*_ *≈* 0.6*T_*x*_) (Fig 2n). Thus, cell collectives on AF apply polarized forces along the direction of collagen alignment (x-direction) which are much lesser in magnitude compared to the directionally unbiased high pulling forces on RF. This further corroborates with our migration track analysis where cells displayed higher persistent motion along collagen alignment in x-direction. This implies, cells pulling unidirectionally along collagen fibers in x-direction need to exert minimum forces orthogonally.

We also compared force propagation across the two conditions, by measuring spatial traction distribution. While tractions were evenly distributed across the monolayer width on AF with forces equivalent to the leading-edge tractions getting exerted at 3/4^th^ distance from edge (1 being the center), there were heterogeneities in RF, with higher tractions localized only to the leading edges (1/4^th^ distance from edge), followed by a steep decrease in middle regions (Fig 2o). This observation suggests that cell-collagen adhesions that exist deeper within the monolayer are getting strongly engaged, indicating a more effective relay of contractile force from the edge to the center on AF compared to RF. Since relay of force occurs via myosin network attached to actin-bundles [47,48], our analysis shows that monolayer on AF utilizes a supracellular network of actin-myosin architecture to relay forces deep within the monolayer. Further, using monolayer stress microscopy [49], we probed these differences by measuring inter-cellular stresses. From the *σ*_*xx*_ kymographs (Fig 2k and 2l), we see significantly lesser build-up of stress at the monolayer midline for AF (0.5*RF) (S2f Fig) parallel to the traction results. To measure stress cooperativity within the monolayer, we calculated the spatial correlation function ($C\left(R\right)={\left(Nvar(\sigma )\right)}^{-1}\sum_{i,j=1}^{N}\left[\sum_{\left|R_{i}-R_{j}\right|}\delta \bar{\sigma_{i}}\delta \bar{\sigma_{j}}\right]$) of average normal stress ($\bar{\sigma }=(\sigma_{max}+\sigma_{min})/2$). Cells on AF exhibited slower spatial decay of *C(R)* (Fig 2p), indicating higher correlation of average normal stresses over greater distances for AF (correlation length higher by 21%). Thus, normal stresses are getting propagated along cell-cell junctions to greater depths within the monolayer on AF. These results are consistent with long-range force transmission seen along cell-cell junctions of collective cells migrating along the length of 3D aligned fibrous matrices [21]. Such fiber driven long-range force transmission phenomenon further allows cells to sense mechanical properties of distant matrices as shown in [20]. Although the stresses experienced by cells on AFs is smaller compared to RF, they are still able to cause faster expansion of monolayer both at the edge and within the monolayer. These results are in contradiction to existing literature which suggest higher forces are needed for faster migration in narrower confinement [18] or aligned nanogroove topographies [32]. Thus, the mechanism governing directional collective migration on aligned fibers is distinct from those previously understood.

### Minimal shear, higher plithotaxis, and monolayer fluidization on aligned fibers explain faster migration

In conventional coupled active matter, including cell monolayers, the extent of cellular mobilization within the collective is governed by transmission of normal stresses across cell coupling junctions. Simultaneously, the local shear landscape and disruption of normal force transmission together determine local flow directions often leading to variegated collective phenotypes such as antiparallel flows [17] and vortices [18]. Since we observe greater mobilization within the monolayer along fiber alignment (AF), we next considered whether this difference between AF and RF stemmed from differences in the extent of agreement between velocities and normal stresses, a phenomenon known as ‘*plithotaxis*’ [15], and shear stresses.

**Fig 3:**
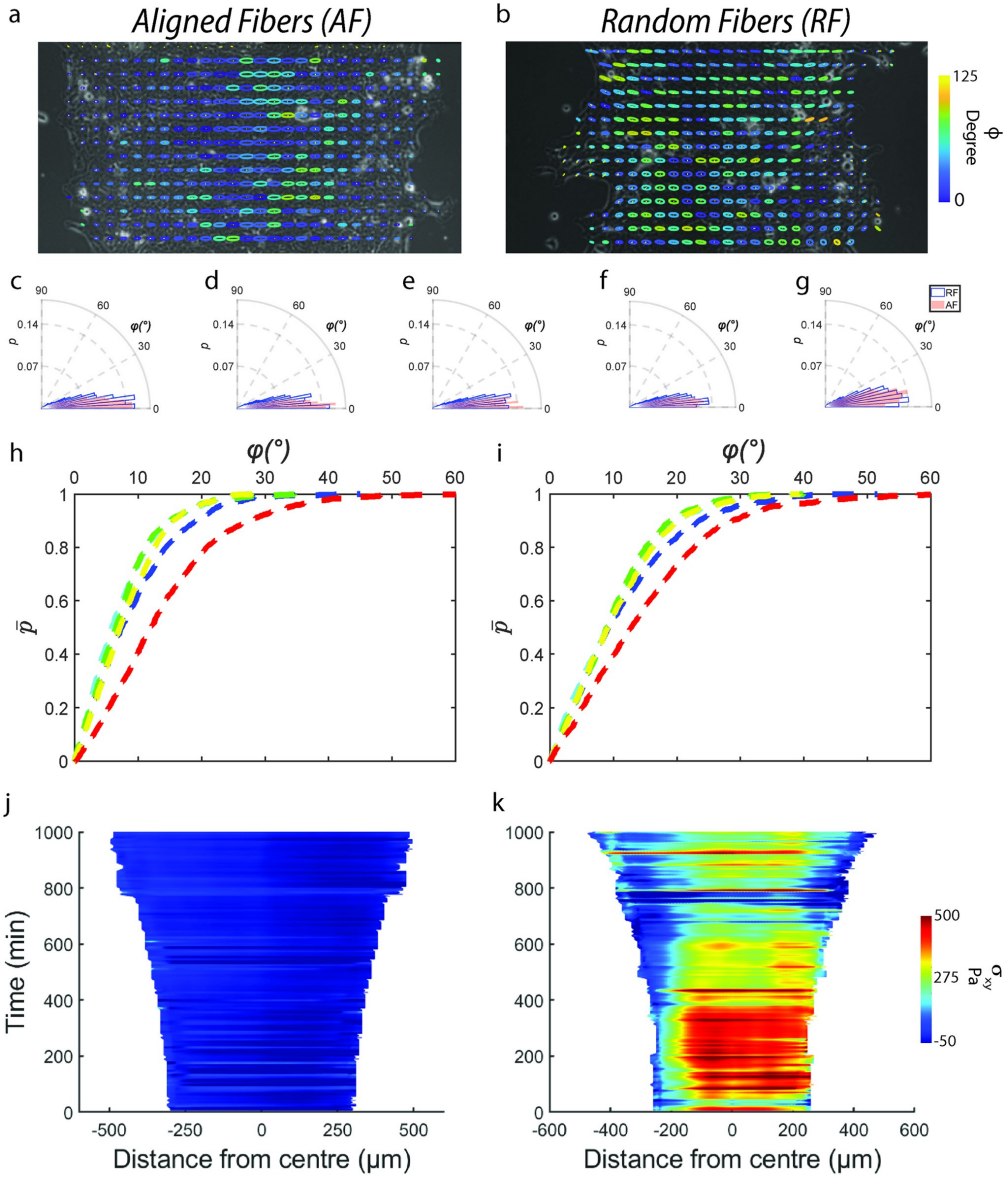
Aligned fibers cause higher *Plithotaxis* within monolayer. Representative monolayer overlaid with Principal stress ellipse which are color-coded for alignment angle *φ* between major axis of principle stress ellipse and direction of cellular velocity on AF (**a**) and RF (**b**). (**c**-**f**) Comparison of alignment angle *φ* distribution between AF and RF for quintiles based on distance from the leading edge with (c) being farthest quintile and (f) being closest quintile. For all quintiles, *φ* distributions are significantly different between AF and RF (p<0.001 for **c-e**, p<0.05 for **f**). Cumulative probability distribution $\bar{P}(\varphi )$ curves (red to blue refers to quintiles at decreasing distance from leading edge, m>1000) for monolayer migrating on AF (**h**) and RF (**i**). Kymographs for monolayer shear stress (*σ*_*xy*_) for AF (**j**) and RF (**k**).

To measure *plithotaxis*, we calculated the angle *φ* between local maximal principal stress (*σ*_*max*_) orientation and local migration velocity vector orientation. We find differences in the extent of *plithotaxis* between AF and RF, as shown by lower *φ* in case of AF compared to RF (Fig 3a and 3b), indicating that stresses are more aligned with velocities in AF. Further, we rank-ordered the pairs of stress and velocity according to their distances from leading edge into quintiles and measured distribution of alignment angle *φ*. We find that for each quintile, the distribution of *φ* is narrower and closer to 0 degrees for AF (Fig 3c–3f). We further calculated cumulative probability distribution of *φ* ($\bar{p}(\varphi )$) for each quintile. Higher alignment would be associated with a steeper rising curve for $\bar{p}(\varphi )$. For both AF and RF, we observe that moving away from the leading-edge causes reduction in alignment between local stress and cellular motion, indicating that *plithotaxis* is most dominant at the leading edge and progressively reduces with increasing distance from leading edge (Fig 3h and 3i). However, for each quintile, AF shows stronger alignment between stress and velocity (steeper $\bar{p}(\varphi )$ curve), indicating more dominant *plithotaxis* compared to RF and suggests that cells are minimizing the generation of shear stress (*σ*_*xy*_) within the monolayer. We confirmed this from the *σ*_*xy*_ kymographs (Fig 3j and 3k) which show that *σ*_*xy*_ is significantly lower on AF.

**Fig 4:**
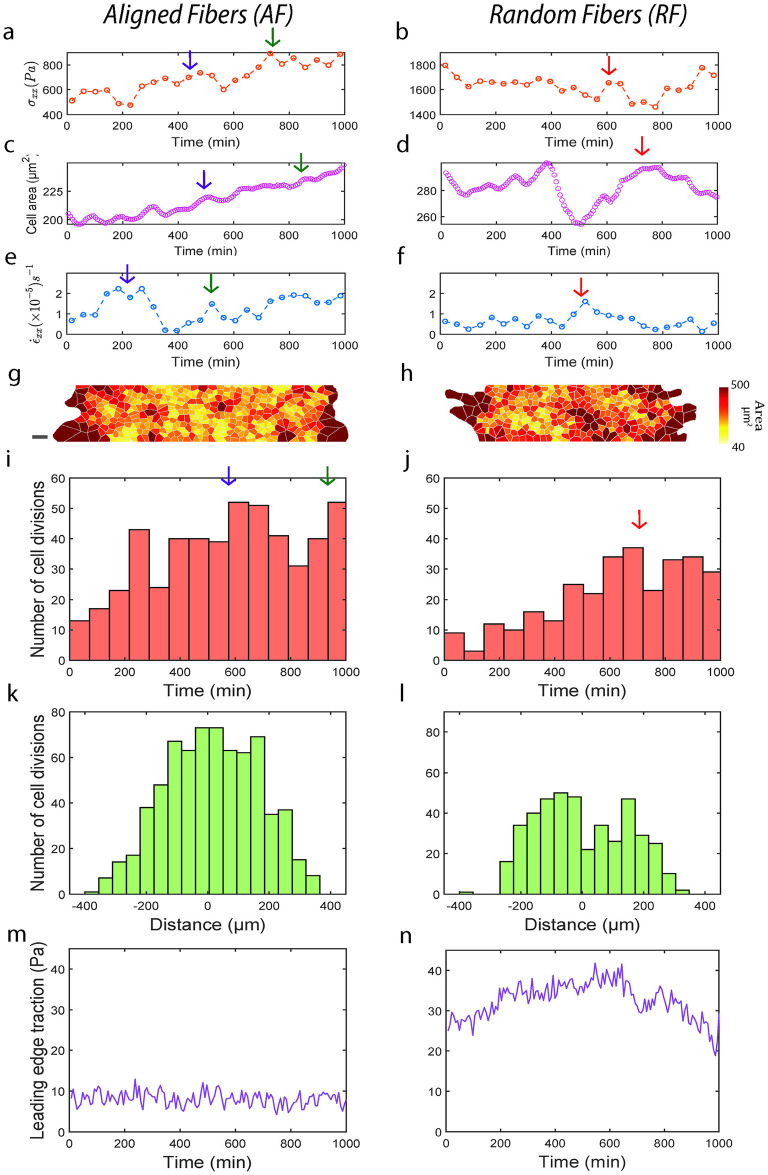
Aligned fibers cause higher fluidization within monolayer. Plot showing temporal evolution of *σ*_*xx*_ (averaged across a 100 μm wide strip at the monolayer midline) on AF (**a**) and RF (**b**). Plot showing temporal evolution of cellular area on AF (**c**) and RF (**d**). Plot showing temporal evolution of ${\dot{\varepsilon }}_{xx}$ on AF (**e**) and RF (**f**). Polygons approximating cell shapes for representative monolayer migrating on AF (**g**) and RF (**h**). Histogram showing temporal evolution of cell division frequency (averaged across a 100 μm wide strip at the monolayer midline) on AF (**i**) and RF (**j**). Histogram showing spatial distribution of cell division frequency (averaged over the entire duration of migration) on AF (**k**) and RF (**l**). Plot showing temporal evolution of leading-edge traction (averaged across 3/4^th^ of the monolayer width) on AF (**m**) and averaged across 1/4^th^ of the monolayer width on RF (**n**).

Previously, cytoskeletal fluidization has been shown to regulate dissipation of built-up stresses within cell monolayers, necessary for their active expansion [45]. Here, we measure such fluidization by measuring stress oscillations (*σ*_*xx*_) at the monolayer midline (100 μm wide strip) (Fig 4a and 4b). Parallelly, we measure cellular area fluctuations (Fig 4c and 4d), since recent work has shown tight coordination of cell size and stress within monolayer via extracellular signal mediated kinase activation [50]. Consistent with the strain-rate fluctuations (discussed in previous section, Fig 4e and 4f), two stress peaks arise for AF within the first 10 hours, compared to a single peak in RF arising after the 10^th^ hour mark (arrows in Fig 4a and 4b). Further, fluctuations in *σ*_*xx*_ were in phase with fluctuations in cellular area (arrows in Fig 4a, 4b and 4c, 4d) and at phase quadrature to strain-rate fluctuations (Fig 4a, 4b and 4e, 4f) [45]. Altogether, a higher frequency of stress dissipation on AF (measured by higher frequency of peaks and valley in temporal stress profile) prevents excessive build-up of stresses within the monolayer, thus explaining why cells on AF experience reduced stresses. Frequent stress dissipation is also tied to frequent regulation of cellular areas which also explains why cell sizes on AF are smaller on AF (Figs 4g, 4h and 1n).

Given that stresses and cellular areas are more frequently regulated in AF, we wanted to investigate the mechanism behind this regulation. Recent literature [51–54] have shown that cellular stretching during monolayer expansion triggers rapid division within 1–2 hours. One such mechanism involves expediting cellular re-entry from early G2 to M-phase via a Ca^+2^ and ERK1/2-dependent cyclin B transcription [53] Thus, we measured the amount of cell divisions that occurred within the monolayer. Looking at the temporal distribution of cell divisions across AF and RF (Fig 4i and 4j), we found that cell divisions on AF were much higher than on RF throughout the 18 hours of migration and that these cell divisions followed the kinetics of cellular area changes (within 3 hours, arrows in Fig 4c, 4d and 4i, 4j). From the spatial distribution of cell division between AF and RF, we found that cell division are maximum at the monolayer midline on AF while on RF, it is roughly 100 μm away from the monolayer center (Fig 4k and 4l). This indicates that stretching induced at the monolayer edge via protrusions travel efficiently via cell-cell junctions all the way to the center leading to maximum cell divisions at the center in AF while on RF, transfer of mechanical stretch at the leading edge breaks down 100 μm away from the monolayer center. This observation runs parallel to our previous results of more efficient velocity and stress propagation in AF compared to RF. The increase in cellular divisions on AF further corroborates our increased cell density results in AF (S2d Fig) and provides an explanation for the observed differences. While increase in cell division over time may decrease cell speeds, there is also a concurrent increase in monolayer area due to increasing cell density by virtue of new cells expanding and thus contributing to increase in the net speed. As density-based spreading and active migration have previously been discussed as coupled phenomena that collectively contribute towards monolayer migration [55, 56], we have not considered the relative contributions of these two-opposing phenomenon on collective cell migration.

We have now shown using three independent measurements on velocity, stress and cell division, that motion is more effectively transferred across the width of the monolayer on AF compared to RF. Mechanistically, we think that AF enables strain rates within monolayer to travel more frequently and deeper within the monolayer on AF, thus transferring intra-cellular stresses deeper leading to frequent stretch-based cell division causing fluidization of the monolayer. As a result, stresses are not allowed to build-up within the monolayer allowing it to migrate at higher velocities. On RF, this chain of strain-rate and stress transfer is not as effective which would then lead to an increase in stress build-up because of infrequent fluidization causing the monolayer to stall [57].

**Fig 5:**
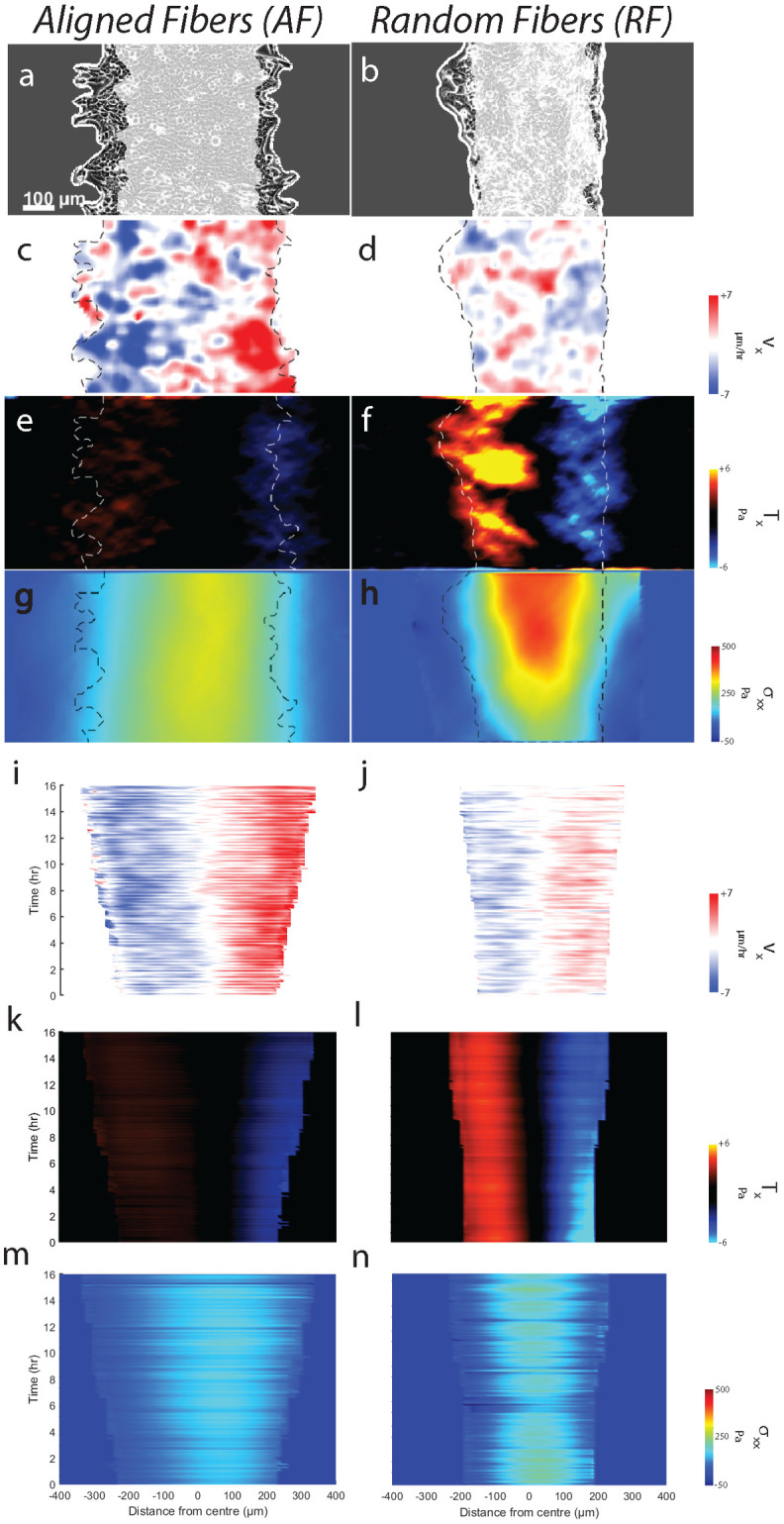
Faster force-effective migration despite reducing substrate stiffness and duration of collagen alignment. Phase contrast images of cell monolayer migrating of softer (**a**) AF and (**b**) RF. (**c**, **d**) Maps of velocity component *v*_*x*_. (**e**, **f**) Maps of traction component *T*_*x*_. (**g**, **h**) Maps of monolayer stress component *σ*_*xx*_. Kymographs of (**i**, **j**) velocity component *v*_*x*_, (**k**, **l**) traction component *T*_*x*_, and (**m**, **n**) monolayer stress component *σ*_*xx*_.

To confirm the above ‘force effective yet fast migration’ phenotype on AF, we repeated the experiments by performing a double negative i.e., further lowering mod-PA substrate stiffness (0.6 kPa) as well as lowering the duration of fiber alignment to 4 hours (from 16 hours). Cells still migrate faster on AF, compared to RF (1.34 times) while applying much lesser strain energies (1/4^th^) on AF compared to RF (Figs 5, S3a and S3c). Both cellular velocities and stresses are correlated across greater distances in clusters migrating on AF showing greater transmission of motion owing to greater force transmission (S3b and S3d Fig). Expectedly, we also observed higher spatial extent of *plithotaxis* in clusters migrating on AF (S3e–S3i Fig).

### Collective motor-clutch model explains ‘force-effective’ collective cell migration via rapid stabilization of cell contractility

**Fig 6:**
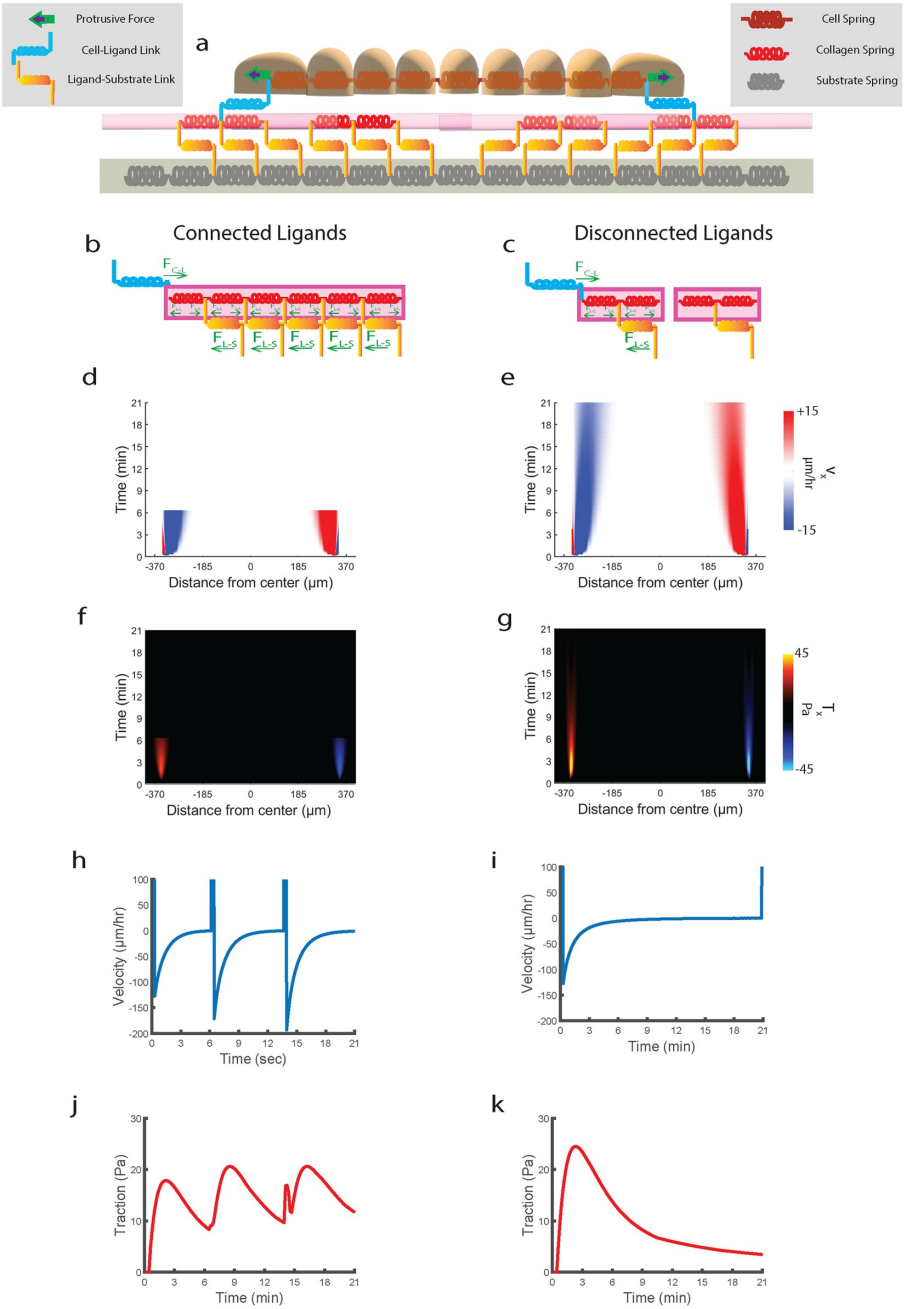
Single contraction simulation shows that ligand connectivity leads to an increase in migration efficiency. (**a**) Schematic of one-dimensional model of epithelial cells represented by springs sliding viscously over rigid ligand springs fixed to substrate springs, after self-propelling forces applied at leader nodes. Schematics showing differences in force balance among cell-ligand forces (*F*_*c-l*_), ligand-ligand forces (*F*_*l-l*_) and ligand-substrate forces (*F*_*l-s*_) between connected ligand (**b**) and disconnected ligand (**c**). Forces are depicted as green arrows. Simulated kymographs of (**d,e**) velocity, (**f,g**) traction for connected (left) and disconnected (right) ligands for single contraction experiment. Plots showing velocity evolution at the leading edge for connected ligand (**h**) during the single contraction event for disconnected ligand (**i**). Plots showing traction evolution at the leading edge for connected ligand (**j**) during the single contraction event for disconnected ligand (**k**).

Our experimental measurements establish that monolayers use aligned collagen fibers to expand faster while generating lesser contractile tractions and monolayer stresses. Also, we show that these forces are transmitted across greater distances within the cluster, leading to higher fluidization and subsequent release of stresses. To understand the governing mechanism behind this force-effective fast migration on AF, we developed a novel motor-clutch model in which the cell monolayer is treated as a series of Kelvin-Voigt viscoelastic springs [45] which is connected to a ligand mesh via clutch-like cell-ECM bonds [58]. The ligand mesh is connected to the substrate mesh via ligand-substrate springs mimicking covalent bonds seen experimentally [33] (Fig 6a). The innovation in our model lies in the ligand mesh sandwiched between cell and substrate meshes which interacts with both via cell-ligand and ligand-substrate bonds respectively. As a result, our model can predict cellular phenotypes based on differences in ligand topography. In our previous paper [33], we have shown that ligands on longer collagen-1 fibers when pulled by an AFM tip, rupture higher number of collagen-substrate covalent bonds compared to shorter fibers. This implies that ligands within the fibers are connected to each other, and longer collagen fibers make greater number of connections to the substrate. Since AF are both longer and aligned compared to RF (S1b–S1e Fig), thus ligands on AF make greater connections to the substrate. Hence, RF is modelled as individual ligand springs disconnected from one another, while AF is modelled by connecting these ligand springs to each other, depicting ligand connectivity along the direction of migration (Fig 6b and 6c). However, for both AF and RF, each substrate node offers a ligand node for binding, thus incorporating similar ligand continuity for both conditions.

Next, to understand whether forces exerted by the contracting monolayer are different across connected and disconnected ligand conditions, we studied cell mesh contraction after a single protrusion event (Fig 6b and 6c). We see that cell mesh contracting on connected ligands comes to rest faster than on disconnected condition (Fig 6d and 6e), while generating lesser traction forces (Fig 6f and 6g). When we repeat protrusion event for the connected-ligands condition, we see that cells can make three protrusion in the same time cells make one protrusion on disconnected ligand condition (Fig 6h and 6i) while also generating lesser traction forces (Fig 6j and 6k). Thus, we show that ligand connectivity increases migration efficiency due to greater engagement of ligand-substrate bonds when cell mesh contracts after a protrusion event, leading to shorter duration for establishing equilibrium and lesser deformation of substrate mesh as well as the higher rate of migration-front stabilization (see methods section ‘*Single Contraction Experiment*’ for mathematical explanation).

**Fig 7:**
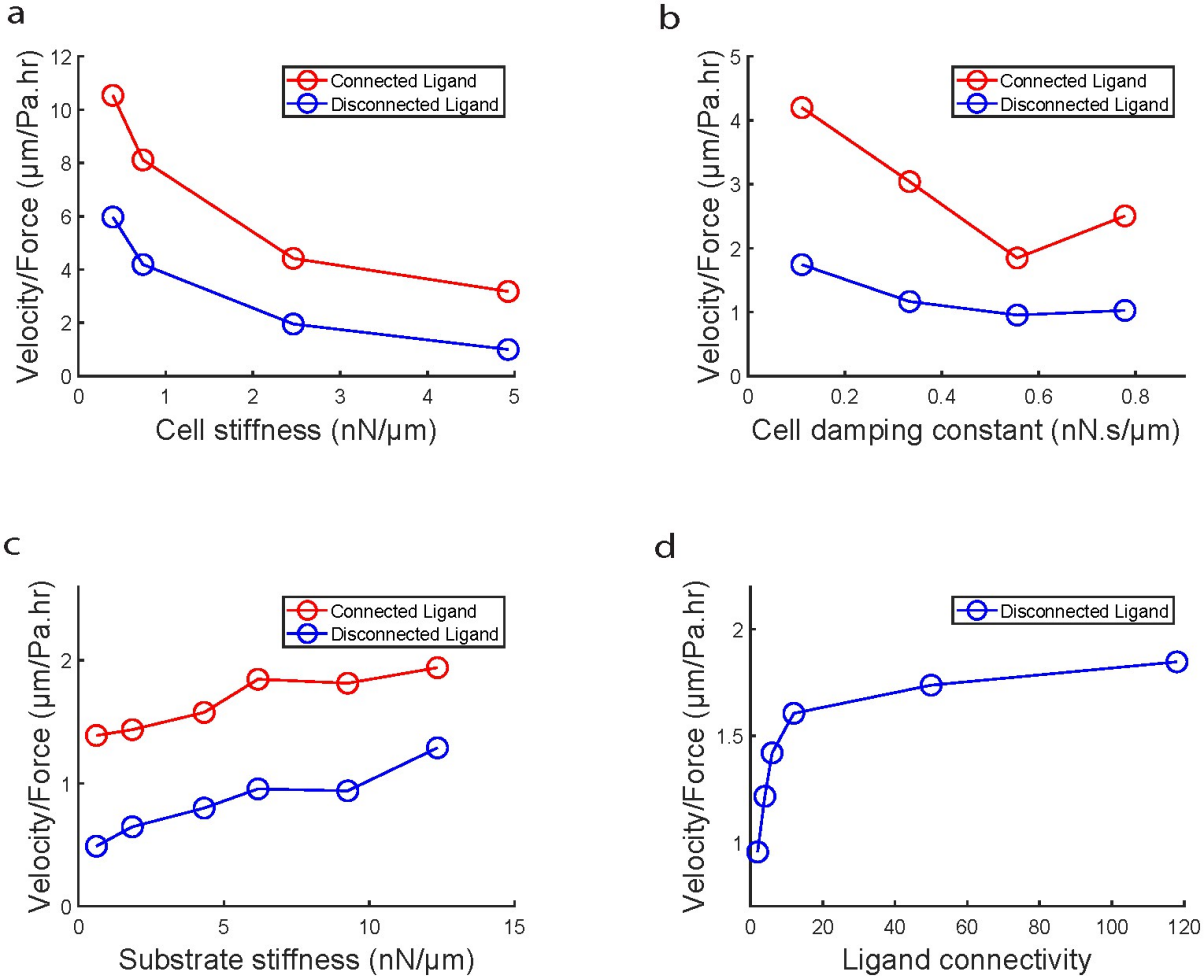
Parameter scan shows migration efficiency is conserved across parameter space. (**a**) Plot comparing migration efficiency (velocity gained/traction exerted) versus cell stiffness across connected and disconnected ligand. (**b**) Plot comparing migration efficiency (velocity gained/traction exerted) versus cell damping constant across connected and disconnected ligand. (**c**) Plot comparing migration efficiency (velocity gained/traction exerted) versus substrate stiffness across connected and disconnected ligand. (**d**) Plot showing evolution of migration efficiency (velocity gained/traction exerted) versus varying ligand connectivity for disconnected ligand condition. Migration efficiency was measured for the initial 5 hours of migration for all data points.

Next, we sought to test whether the observed increase in ligand connectivity mediated increase in migration efficiency held true across a range of values for the various model parameters. We first varied the stiffness of the cell springs (*k*_*c*_) and found that for all the stiffnesses tested, connected ligand condition resulted in higher migration efficiency compared to disconnected ligands (Fig 7a). However, we did observe that increasing *k*_*c*_ lead to decrease in migration efficiency. Since stiffness of cell springs is a measure of cell contractility [59, 60], this plot shows that increasing contractility without increasing protrusive forces can negatively affect collective migration efficiency implying that there needs to be balance between protrusive and contractile forces for effective collective migration [61]. Next, we varied cellular damping constant (*η*_*c*_) and observed across the range of *η*_*c*_, migration on connected ligands was more efficient than disconnected ligand condition (Fig 7b). Additionally, we observed that increasing *η*_*c*_ reduces migration efficiency, implying that increase in friction due to maturing cell-cell contacts leads to jammed amorphous motion [62]. Next, we varied substrate stiffness by varying the stiffnesses of individual springs (*k*_*s*_) comprising the substrate mesh (Fig 7c) and we observed that for all tested values of *k*_*s*_, connected ligand condition displayed higher migration efficiency compared to disconnected condition. We also observe that increasing substrate stiffness increases migration efficiency [63]. We also looked at the effect of ligand connectivity on migration efficiency by varying the extent of connectivity in disconnected condition by varying the number of ligand nodes that were connected to each other. We observe that increasing ligand connectivity increases migration efficiency (Fig 7d).

**Fig 8:**
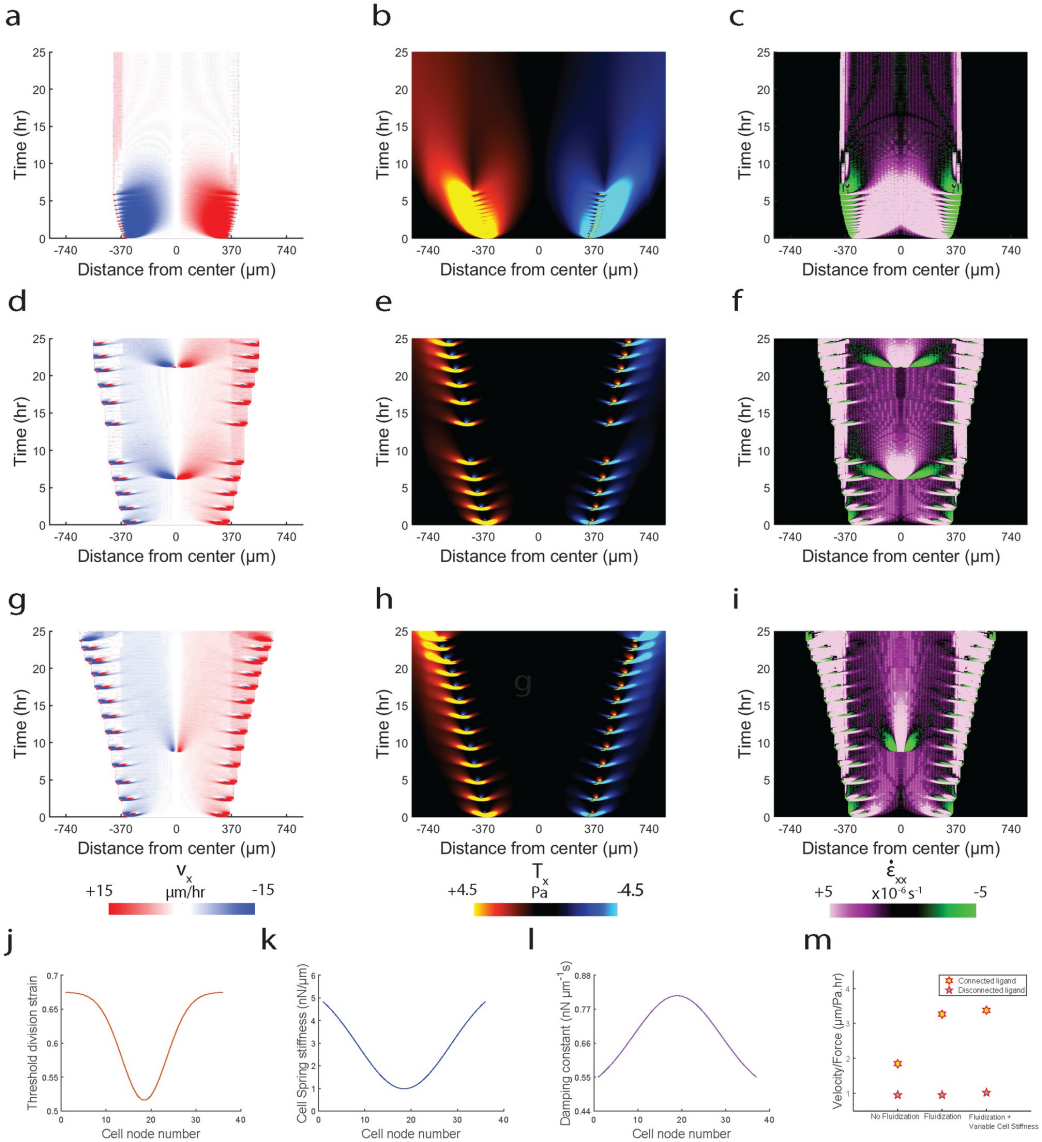
Fluidization within monolayer further increases migration efficiency. Simulated kymographs of (**a**) velocity, (**b**) traction and (**c**) strain-rate for connected ligand condition without fluidization. Simulated kymographs of (**d**) velocity, (**e**) traction and (**f**) strain-rate for connected ligand condition with fluidization. Simulated kymographs of (**g**) velocity, (**h**) traction and (**i**) strain-rate for connected ligand condition with fluidization and spatial variation in cell stiffness and damping constant. Plot showing spatial variation in threshold division strain across the cell nodes in silico (**j**). Plot showing spatial variation in spring stiffness across the cell nodes in silico (**k**). Plot showing spatial variation in damping constant across the cell nodes in silico (**l**). Plot comparing migration efficiency (velocity gained/traction exerted) for ‘*no fluidization’*, ‘*fluidization’* and ‘*fluidization with spatial variation in stiffness and damping’* across connected and disconnected ligand conditions (**m**).

In our model, we see that the monolayer starts to stall after ~5 hours of migration (Fig 8a–8c). Parallel to our experimental findings of cell division based monolayer fluidization improving migration efficiency (Fig 4), we also implemented cell division-based fluidization in our model such that cells divide when they reach a threshold stretch (${\varepsilon_{c}}_{th}$). In addition, we implemented a spatial variation in ${\varepsilon_{c}}_{th}$ (Fig 8j) based on our experimental observation that cell divisions occur most frequently near the middle of the monolayer rather than at the edges (Fig 4k and 4l). Upon implementation, we observe that monolayer does not get stalled any more (Fig 8d–8f), but the leading-edge has a convex shape indicating that with progression of time, protrusive forces produce diminished displacements at the leading-edge because the combined contractile forces due to expanded cell springs within the mesh which indicates increase in cellular contractility and responsible for monolayer contraction, starts dominating the protrusive forces at the leading edge which is responsible for monolayer expansion. This anomaly to experimental observations is addressed by inclusion of spatial variations in cellular stiffness (*k*_*c*_) and damping (*η*_*c*_) (Fig 8k–8l and S2 Table). We implemented a spatially varying *k*_*c*_ (Fig 8k) since recent experiments have shown that leader cells have higher stiffness compared to cells in the middle of the monolayer [64]. Further, we implemented spatial variation in *η*_*c*_ (Fig 8l)) inverse to the variation in *k*_*c*_ following the rationale that stiffer cells would be more elastic owing to higher actin organization compared to softer cells [65]. We see that upon implementation of the spatial variations in *k*_*c*_ and *η*_*c*_, the shape of the leading edge is now concave (Fig 8g–8i) as seen experimentally.

**Fig 9:**
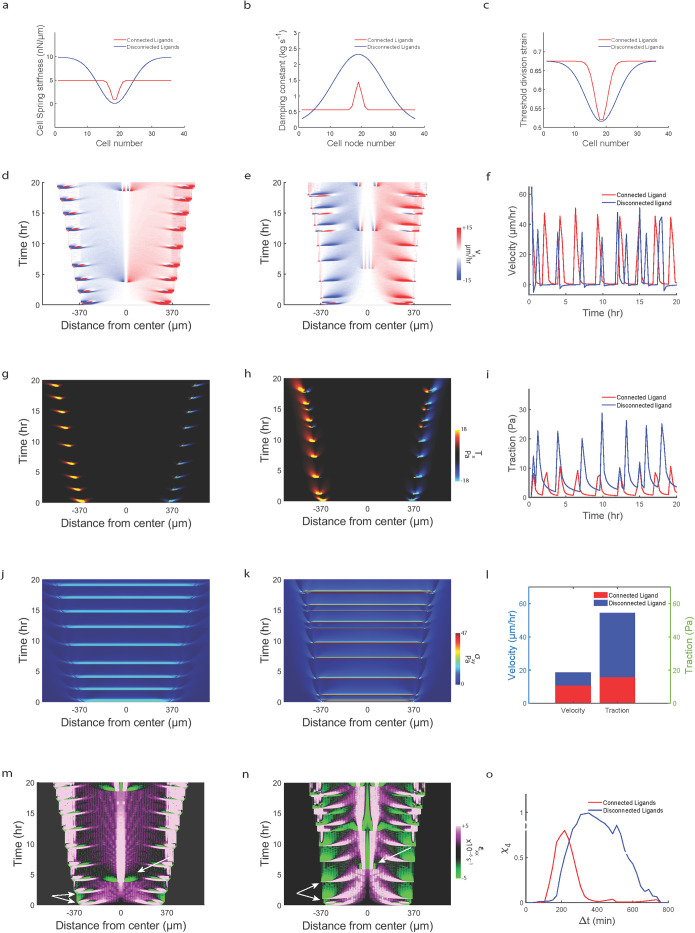
Physical model showing force-effective fast migration due to rapid contractility stabilization by fiber connectivity. Plot comparing spatial variation in spring stiffness across the cells between connected and disconnected ligand conditions (**a**). Plot comparing spatial variation in damping constant across the cell nodes between connected and disconnected ligand conditions (**b**). Plot comparing spatial variation in threshold division strain across the cells between connected and disconnected ligand conditions (**c**). Simulated kymographs of (**d**, **e**) velocity, (**g**, **h**) traction, (**j**, **k**) shear-stress and (**m**, **n**) strain-rate kymograph for connected (left) and disconnected (right) ligands. Plot comparing temporal evolution of leading-edge velocity (**f**) and traction (**i**) between connected and disconnected condition. Plot comparing average velocity and traction between connected and disconnected condition (**l**). Plot comparing four-point susceptibility (*χ*_4_) versus Δ*t* for simulated results (**o**).

We next compare the simulations which include fluidization and spatially varying *k*_*c*_, *η*_*c*_ and ${\varepsilon_{c}}_{th}$ between connected and disconnected ligand condition (S4 Fig). We see that cells on connected ligands migrate faster (*1.7 times) (S4a–S4c Fig) while applying lesser (*1.8 times) traction forces (S4d–S4f Fig) than disconnected ligand condition. However, in experiments we had observed that cells on AF travel 1.56–1.33 times faster (S2a and S3a Figs), while applying 2–3 times lower forces than RF (Fig 2o). Furthermore, the strain-rate waves reach center first on disconnected condition (S4j and S4k Fig), which is inconsistent with experiments where the X-waves reach midline first on AF (Fig 4e and 4f). Additionally, we also observe that although χ_4_ peak (S4l Fig) is predicting unjammed motion for connected condition, it is not congruent with experimental χ_4_ peaks of AF and RF (Fig 1n). To address these shortcomings in our model, next we decided to include differences in actin-myosin network distribution across AF and RF which we inferred from traction distribution differences between AF and RF (Fig 2o), by incorporating different spatial variations in *k*_*c*_ (*x*_*c*_), *η*_*c*_(*x*_*c*_) and ${\varepsilon_{c}}_{th}(x_{c})$ for connected and disconnected conditions (Fig 9a–9c). Since in our model, stiffness of the cell springs (*k*_*c*_) is a measure of myosin driven contractility, we implemented differences in spatial variation of *k*_*c*_ between connected and disconnected ligand condition (Fig 9a) by mimicking difference in the monolayer width dependent traction profile seen between AF and RF respectively (Fig 2o). Consequently, we implemented differences in spatial variation of *η*_*c*_ (Fig 9b) following the assumption that uniform spatial cellular stiffness (*k*_*c*_) on connected ligand condition (Fig 9a) would imply uniform spatial cellular viscosity (*η*_*c*_) while on disconnected ligand condition high *k*_*c*_ at the monolayer edges would mean low *η*_*c*_ at the edges and low *k*_*c*_ at the monolayer center would mean high *η*_*c*_ at the center [65]. Additionally, we also incorporate differences in spatial variation of threshold division strain (${\varepsilon_{c}}_{th}(x_{c})$) between connected and disconnected ligand condition (Fig 9c) following our observation of differences in position of maximum cell divisions between AF and RF (Fig 4k and 4l) and previous literature showing that cell stiffness modulates cell-division frequency [66,67].

As a result, our model correctly predicts an increase in velocity (*1.38 times) in parallel with a decrease (*2.42 times) in traction forces for cells migrating on connected ligands compared to disconnected condition (Fig 9l). Our model also correctly predicts an increase in monolayer fluidization (white arrows in Fig 9m and 9n) with an increase in ligand connectivity along with faster and deeper transfer of strain rate waves within the cell mesh (Fig 9m and 9n). As a result, unjamming of motion emerges, as shown by the shift in χ_4_ peak to shorter time scales and reduction in peak height (Fig 9o), which are remarkably consistent with experiments (Fig 1n) when compared to the disconnected condition.

**Fig 10:**
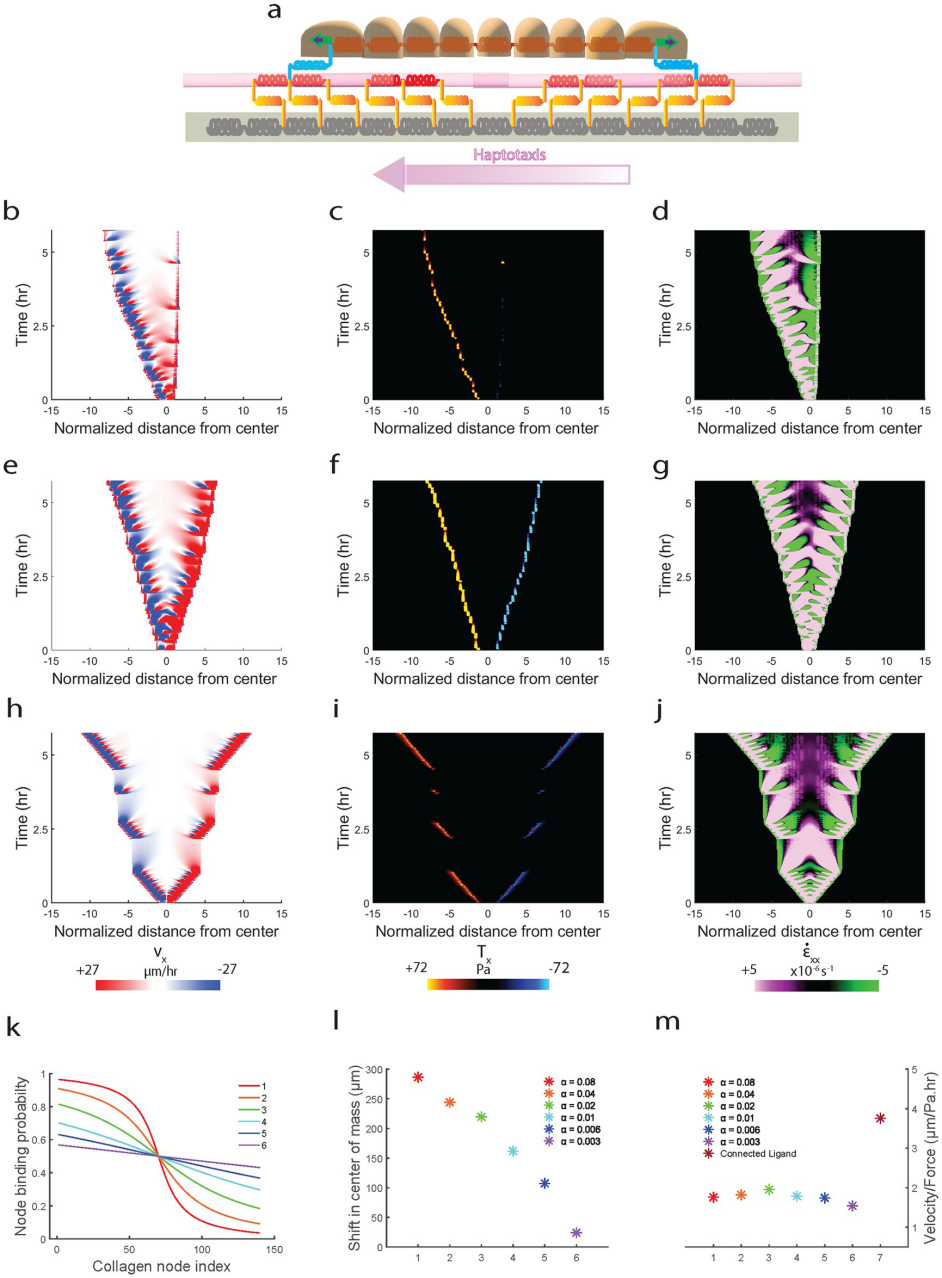
Model capturing the phenomenon of *Haptotaxis* and depicting ligand connectivity to be superior in terms of migration efficiency when compared to ligand continuity. (**a**) Schematic of one-dimensional model showing direction of increasing ligand binding probability (*ρ*_*l*_) from right-to-left, discussed further in supplementary section. Simulated kymographs for (**b**) velocity, (**c**) traction and (**d**) strain-rate for *α* = 0.04. Simulated kymographs for (**e**) velocity, (**f**) traction and (**g**) strain-rate for *α* = 0.003. Simulated kymographs for (**h**) velocity, (**i**) traction and (**j**) strain-rate for connected ligand condition. Plot showing spatial variation of ligand binding probability (*ρ*_*l*_(*x*_c_)) for different values of *α* (**k**). Plot showing shift in center of mass of the cell mesh migrating on ligand mesh having different *ρ*_*l*_(*x*_c_) corresponding to different values of *α* (**l**). Plot comparing migration efficiency for cell mesh migrating on ligand mesh having different values of *α* (shown in plot k) (**m**).

Thus far, we have studied the effects of changing ligand connectivity on migration efficiency while keeping ligand continuity constant across the conditions. Next, we ask whether changing ligand continuity with constant connectivity has similar effects on migration efficiency. To this end, we implemented the phenomenon of *haptotaxis* using our model (Fig 10). We implemented a gradient in ligand binding probability (increasing right to left, Fig 10a) which restricts binding ability of cell mesh nodes to ligand nodes (Eq 13, methods section), thus mimicking a gradient in ligand concentration along direction of migration (see methods section ‘*Implementing ‘Haptotaxis*” for implementation details). We clearly see the higher shift in cell monolayer center of mass for steepest gradient (Fig 10b–10d) compared to intermediate ligand binding gradient (Fig 10e–10g) and zero gradient (Fig 10h–10j). Indeed, for all the different ligand binding gradients that we tested (Fig 10k), we observe that steeper gradients lead to higher shift in center of mass indicating higher *haptotaxis* (Fig 10l). However, higher gradients in ligand binding probability do not necessarily equate to higher migration efficiency, with the highest migration efficiency occurring at an intermediate ligand binding gradient (Fig 10m) [68–70]. Additionally, for all the gradients tested, migration efficiency is still lower than that on connected ligand condition, indicating that ligand connectivity is more potent in terms of affecting migration efficiency compared to ligand continuity. Moreover, we also measured migration efficiency with varying ligand density and found that at both high and low densities, connected ligand condition has higher migration efficiency than disconnected condition (S5 Fig).

**Fig 11:**
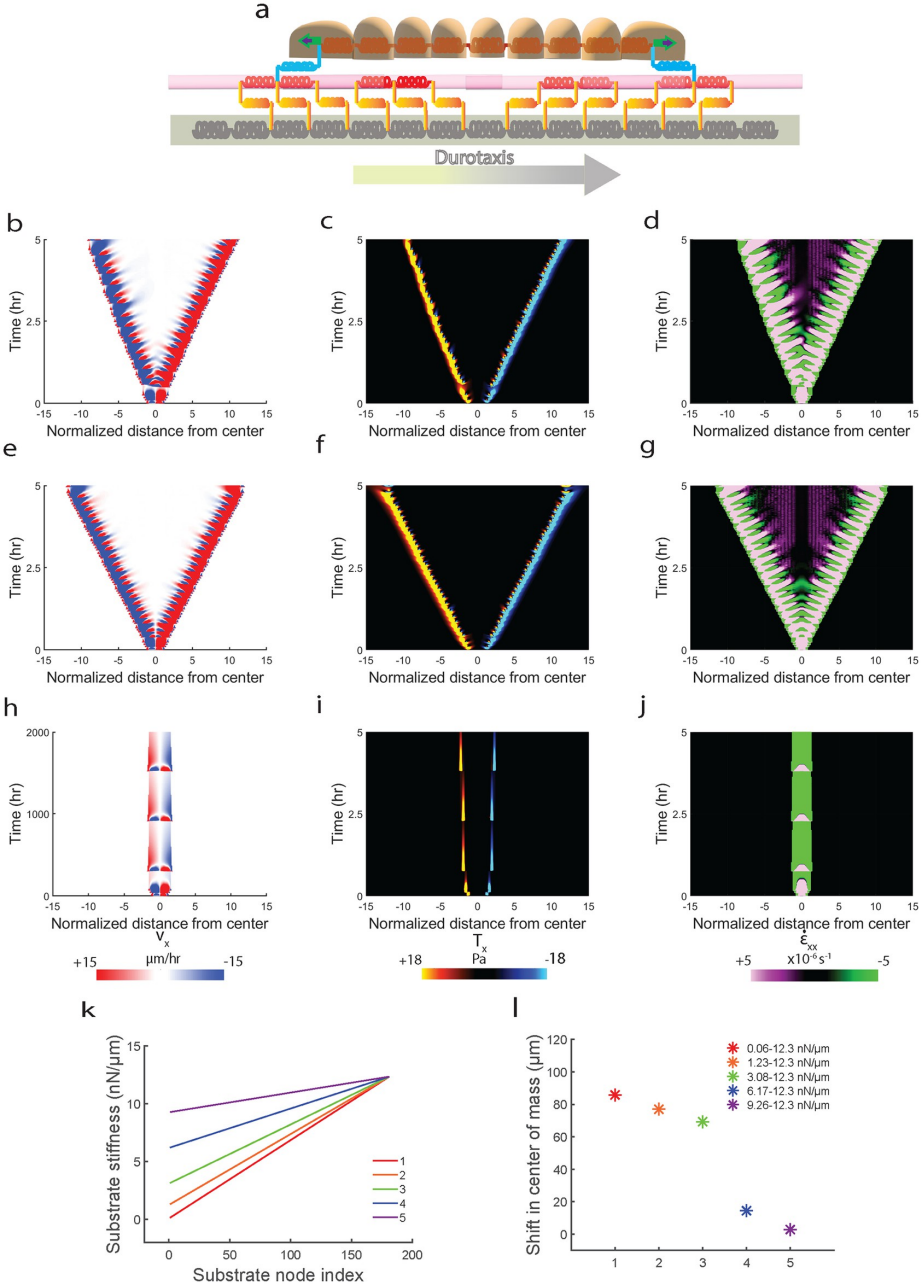
Model capturing the phenomenon of *Durotaxis* and showing higher rate of contractility stabilization is sufficient to capture the phenomenon. (**a**) Schematic of one-dimensional model showing direction of increasing substrate stiffness (*k*_*s*_) from left-to-right, discussed further in supplementary section. Simulated kymographs for (**b**) velocity, (**c**) traction and (**d**) strain-rate for *durotaxis* stiffness range of 0.06 *nN*/*μm* < *k*_*s*_ < 12.3 *nN*/*μm*. Simulated kymographs for (**e**) velocity, (**f**) traction and (**g**) strain-rate for uniformly stiff substrates (*k*_*s*_ = 12.3 *nN*/*μm*). Simulated kymographs for (**h**) velocity, (**i**) traction and (**j**) strain-rate for uniformly soft substrates (*k*_*s*_ = 0.06 *nN*/*μm*). Plot showing different spatial variation of substrate stiffness (*k*_*s*_(*x*_*s*_)) used for testing the *durotaxis* model (**k**). Plot showing shift in center of mass of the cell mesh migrating on substrate mesh having different variations in *k*_*s*_(*x*_*s*_) (shown in plot k) (**l**).

Next, we sought to test whether our model could be further generalized to predict other known forms of directional migration. To this end, we decided to implement *durotaxis*–a form of directional migration towards increasing substrate stiffness, phenomenologically using our motor-clutch model. We implemented a linear gradient in substrate spring stiffness (*k*_*s*_) (Eq 14, methods section) with *k*_*s*_ increasing from left-to-right (Fig 11a) (see methods section ‘*Implementing ‘Durotaxis*” for implementation details). We observe that implementing gradient in stiffness causes cell mesh to undergo a shift in center of mass towards increasing stiffness (Fig 11b–11d), while cell mesh migrating on uniformly stiff (Fig 11e–11g) and soft substrates (Fig 11h–11j) expand uniformly without shift in center of mass although monolayer expansion on uniformly stiff substrates is much higher than uniformly soft substrates [63]. We next tested the extent of *durotaxis* on varying stiffness gradients (Fig 11k) and found the stiffer gradients in stiffnesses displayed higher *durotaxis* (higher shift in center of mass) (Fig 11l) [28].

## Discussion

Given close agreement between experiments and model predictions, we conclude that increased frictional resistance via parallel engagement of connected ligands enables a new, efficient mode of migration wherein lower net forces generate faster cell migration on matrices that offer higher ligand connectivity. This hitherto unknown mode of efficient migration is counterintuitive to the traditional understanding of proportional force-speed relationship in collective migration [18,31,32]. While cell-cell force propagation has been shown to regulate collective migration [28], our experiments use constant matrix stiffness across conditions, thus controlling for cellular mechanotransduction, and elucidate how matrix fibers cause supracellular directional migration through long-range extracellular force transmission. We also speculate that utilizing aligned ECM fibers on 2D soft substrates gives rise to a quasi-2D mode of contact-guided directional migration. In this mode, cells utilize the connectivity of ligands offered by aligned fibers, thus delineating the role of ligand connectivity from ligand continuity presented by uniformly coated ligands on micropatterned protein stripes or topographical grooves [17,18].

As evident from our experiments, uniform ligand coating of random collagen fibers leads to ligand continuity but not connectivity, which restricts the frictional resistance offered by ligand substrate bonds which do not get parallelly engaged as seen in case of aligned fibers. Similar phenomenon has been observed in single cells when they experience changes in ligand continuity [71], where single cells migrate faster yet apply lower forces when they experience ligand continuity compared to sharp decrease in ligand continuity. At the scale of a single cell, short fibers offer higher ligand connectivity compared to abrupt break in ligand continuity. This rise in migration efficiency mediated by aligned fiber has direct implications in cancer metastasis where tumor microenvironment presents collagen fibers of varying alignment concomitant with cancer progression. For example, recent work has shown that cell collectives use significantly less ATPs while migrating on aligned fibers compared to randomly oriented fibers [72], while breast tumor histology revealing aligned fibers offers poor prognosis [35], implying that tumor cells align matrix fibers to metastasize in an energy efficient manner. Our work reveals a previously unexplored dimension of ligand connectivity and its connection to collective migration efficiency. These findings present a new paradigm of migration efficiency regulated not just by force but by the rate of migration front stability. While only recently studies have shown the importance of migration front stability in dictating directional migration [29,30], we show that a higher rate of front stability leads to more efficient migration and further show that this phenomenon is enough to capture *durotaxis*, a known form of efficient directional migration.

## Materials and methods

### Cell culture

MCF10A cells (Tissue Culture Support Center, Washington University, St Louis, USA) were cultured in DMEM/F12 (Invitrogen) supplemented with 5% (v/v) horse serum (Invitrogen), 20 ng/ml epidermal growth factor (EGF, Miltenyi Biotec Inc), 0.5 mg/ml hydrocortisone (Sigma-Aldrich), 100 ng/ml cholera toxin (Sigma-Aldrich), 10 μl/ml insulin (Sigma-Aldrich) and 1% (v/v) penicillin-streptomycin (Sigma-Aldrich).

### Microfabrication of magnetic PDMS stencils

Cell monolayer was confined to a particular geometry by depositing magnetic PDMS stencil with a rectangular opening (500 μm) over the gel (S1 Fig) [13]. Briefly, a mixture of PDMS and magnetite (25% w/w) was added to a mold which was 3D printed (Proto Labs Inc.). The mold containing PDMS mixture was kept in an oven to bake at 80° C. A magnet was attached to the bottom of the well plate just below the PA gels so that it could secure the magnetic PDMS stencil on top of the wet PA gel.

### Fabrication of mod–PA hydrogels

Polyacrylamide (PA) gels were chemically modified by a method previously described in [33]. Briefly, N-hydroxyethyl acrylamide (HEA) was oxidized to its primary aldehyde group N-ethanal acrylamide (EA) using pyridinium chlorochromate (PCC). The crude oxidized product was first purified via vacuum filtration to remove larger impurities and further purified by column chromatography using silica as solid phase and Ethyl Acetate as the mobile phase. Briefly, crude product was dissolved in Chloroform before running it through the column to separate product which was obtained as a white flakey solid upon evaporating excess solvent in a rota-evaporator (Buchi). Fourier-transform infrared spectroscopy (FTIR) was performed to confirm the presence of aldehyde group in the purified product (S1f Fig).

The synthesized EA was then incorporated into PA to facilitate protein conjugation. Modified Polyacrylamide gels of desired stiffness were prepared on glass-bottom 6 well-plates (Cellvis). Precursor PA solution was prepared by mixing acrylamide (A, Bio-Rad), EA and bis-acrylamide (B, Bio-Rad) (2.8% A:2.8% EA: 0.44% B) to result in soft gels (approximate stiffness of 2.4 kPa) as reported previously [73]. Softer gels were synthesized using recipe (4.592% A:1.0% EA, 0.44% B) to result in approximate stiffness of 0.3 kPa [33]. Polymerization was initiated by adding 0.5% ammonium persulfate (APS, Sigma-Aldrich) and 0.05% tetramethylethylenediamine (TEMED, Sigma-Aldrich) to the precursor mixture. Then, 60 μL of the precursor solution is sandwiched between a hydrophobic cover slip treated with Sigmacote (Millipore) and the silanized glass bottom 6 well plates. Next, the solution was allowed to polymerize in a vacuum chamber for 45 mins.

### Magnetic alignment of Collagen-1

The modified PA gels were then incubated in 0.1 mg ml^-1^ type 1 collagen (rat tail collagen, Santa Cruz Biotechnology) inside the bore of a horizontal bore electromagnet for 2 hours at 4 C while fibillogenesis took place. Field strength for the magnet was 11 T (S1 Fig). Collagen fibrils aligned at an angle of 90° to the direction of magnetic field due to presence of a positive diamagnetic anisotropy of the growing collagen fibrils [74,75]. The aligned fibrils deposited onto the gel and formed covalent linkages with the modified PA gel as described previously [33].

For gels with uniform collagen deposition, modified PA gels were incubated with 0.1 mg ml^-1^ type 1 collagen at 37 C for 1 h.

### Fluorescent labelling of collagen and imaging

Collagen was fluorescently labelled according to method described in [76]. Briefly, 1.5 ml of a 3 mg/ml collagen 1 solution (pH ~7.5) was gelled at 37°C inside a 12 multiwell plate and incubated with 0.2 M sodium bicarbonate buffer (pH 9.0, Sigma-Aldrich) for 10 min. Next, gel was incubated in 500 μl of Sulfo-Cyanine5 NHS ester dye (Lumiprobe Co. USA) solution (in DMSO) at room temperature in dark for 1 h. Remaining NHS ester was quenched by adding 3 ml of 50 mM Tris-HCL buffer (pH 7.5, Sigma-Aldrich). Later, stained collagen gel was washed with PBS 6 times over a period of 2 h. Afterwards, labelled gel was solubilized using 200 mM HCl (Sigma-Aldrich). The solubilized collagen solution was dialyzed against 20 mM acetic acid (Sigma-Aldrich) at a 1:1000 ratio with continuous stirring at 4°C for 4 h. Working collagen solution was prepared by replacing 4% of the unlabeled collagen solution with its labelled counterpart. Collagen coated hydrogels were imaged using Zeiss LSM 880 laser confocal microscope (Carl Zeiss Microscopy, Germany) (S1b and S1c Fig).

### Cell monolayer patterning on soft hydrogels

Prior to using PDMS stencils for patterning, they were stored in 70% ethanol overnight. To prevent cell and ligand adhesion to PDMS, stencils were then passivated by incubating them in 2% Pluronic F-127 (Sigma-Aldrich) dissolved in PBS for 1 h. During the same time, gels which were coated with collagen are washed twice in PBS and incubated with media at 37° C. Magnets were attached to the bottom of the glass bottom dishes just below the hydrogels. Passivated PDMS stencils were washed twice in PBS and air dried for 20 min, and then were deposited on the surface of the hydrogels. Next, 100 μl of media containing 60,000 cells was added into the exposed region of the hydrogel and kept inside incubator for the cells to attach. After 1-hour, unattached cells were washed away and 100 μl of fresh media was added. Twelve hours after seeding, cells reached desired cell density and stencils were carefully removed using tweezers and 3 ml of fresh media was added onto the wells.

### Time-lapse microscopy

Migrating cells were imaged using Zeiss AxioObserver Z1 microscope (Carl Zeiss Microscopy) fitted with incubation. Cells were imaged after a 7 min interval over a period of 20 h using a 10x objective. Cells were allowed to acclimatize to the microscope incubation for 3 h before beginning image acquisition. Experiments were conducted in 37° C and 5% CO_2_.To include the entire width of the monolayer, two images with 10% overlap were captured and then stitched using Zeiss stitching tool.

### Cell velocities and trajectories

Velocity fields for migrating cells were computed via particle image velocimetry (PIV) though PIVlab package in MATLAB [77]. Source code was modified to include a two-dimensional Hanning window across the interrogation window to improve PIV resolution and prevent boundary artifacts during cross-correlation. Velocity field (*v*_*ij*_) was obtained by running PIV for three passes of 64-, 32- and 16- pixel windows. Monolayer boundaries were computed using home-built MATLAB script. Cell trajectories were obtained by tracking labelled cell nuclei using Fiji plugin TrackMate. Cell trajectories were imported in MATLAB and further analyzed using custom written scripts. Average cellular persistence in motion was measured as $\bar{p}=\langle \frac{L_{i,e}}{L_{i,c}}\rangle$, where *L*_*i*,*e*_ is Euclidean distance covered by the cell *i* and *L*_*i*,*c*_ is the contour length of its track. 〈…〉 denotes average over all such tracks.

### Quantifying dynamics of cellular motion

To quantify dynamics of cellular motion, we calculated the self-overlap order parameter [78].

$$Q\left(\Delta t\right)=N^{-1}\sum_{i=1}^{N}w_{i}$$

Where *N* is the number of cells, *w* = 1 if $\left|r_{i}\left(t+\Delta t\right)-r_{i}\left(t\right)\right|<0.9*d_{c}$ (where *r*_*i*_(*t*) is x-position of cell at time *t* and *d*_*c*_ is average cell diameter) and *w* = 0 otherwise. To measure the size and lifetime of the cooperative fluctuations within the migrating cluster of cells [79], the spatially heterogeneous dynamics in cellular motion was quantified by the four-point susceptibility χ_4_, given by

$$\chi_{4}\left(\Delta t\right)=N\left[\langle Q{\left(\Delta t\right)}^{2}\rangle -{\langle Q(\Delta t)\rangle }^{2}\right]$$

Where *N* is the number of cells, 〈…〉 shows the average over sequence of images at all times, *t*. χ_4_ (Δ*t*), is the four-point susceptibility and quantitatively shows the size of cooperatively motile cell clusters and their associated rearrangement times.

### Fourier-transform traction microscopy

Traction forces were calculated from gel deformations which were recorded by measuring displacements in fluorescent marker beads (0.2 μm, Invitrogen) embedded inside the hydrogels on which cells were migrating. Displacements of the beads were measured in reference to the initial undisturbed condition obtained after trypsinization (10x) of cells. Bead strain field was calculated using PIV. This strain was then used to calculate the traction field by solving the inverse problem using a non-regularized Fourier transform algorithm previously employed in [4]. The algorithm was implemented in MATLAB. The total energy *U* contribution from the monolayer towards the elastic distortion of the substrate was calculated by [80]

$$U=(\frac{1}{2})\int \vec{T}\left(\vec{r}\right).\vec{u}\left(\vec{r}\right)dxdy$$

### Monolayer stress microscopy

Stresses within the monolayer were calculated using monolayer stress microscopy implemented in MATLAB [49]. $\sigma_{ij}=\left(K_{1}-\frac{2}{3K_{2}}\right){\dot{\varepsilon }}_{kk}\delta_{ij}+2K_{2}{\dot{\varepsilon }}_{ij},\;K_{1}$ and *K*_2_ are bulk and shear viscosities, ${\dot{\varepsilon }}_{ij}$ is strain-rate tensor. Finite element scheme was used to model cell monolayer, assumed to behave as thin sheet of uniform thickness (Poisson’s ration 0.5, 5 μm height). Boundary conditions at the leading edge were assumed to be stress free. At the top and bottom optical edges, normal displacements and shear stresses were assumed to be zero (*u*_*i*_*n*_*i*_ = 0, *σ*_*ij*_*n*_*j*_*t*_*i*_ = 0) [81].

### Spatial autocorrelation function of tension

Extent of spatial cell-cell coordination in terms of velocities and stresses was quantified by the spatial autocorrelation function [15]

$$C\left(R\right)=\frac{1}{Nvar(\bar{\sigma })}\sum_{i,j=1}^{N}\left[\sum_{\left|R_{i}-R_{j}\right|=R}\delta {\bar{\sigma }}_{i}\delta {\bar{\sigma }}_{i}\right]$$

$\delta {\bar{\sigma }}_{i}$ is local departure of tension at position *R*_*i*_ from the spatial mean 〈*σ*〉, while $var(\bar{\sigma })$ is the variance of those departures, and |*R*_*i*_–*R*_*j*_| = *R* denotes the bin width which consists of *N* points. We used the same function to quantify the spatial coordination of velocity vectors. Plots were generated by averaging 4 consecutive values for smoothening purposes.

### Kymographs

Every pixel’s position within the monolayer was computed from the nearest leading edge. Next, we calculated mean values of velocities, velocity strain rates, tractions and monolayer stresses of all pixels at a given distance from leading edge. These values were then represented as a single dimension segment of the monolayer for a single time point. These segments were obtained for every time point and staked together to result in the kymographs.

### Quantifying cell shapes

900 cells for AF and 600 cells for RF were manually tracked over 185 frames by annotating their nuclei using MATLAB image labelling app. Labelled cell nuclei were then used to generate a Voronoi tessellation to approximate cellular boundaries. These boundaries were used to approximate the area of cells. The cell’s aspect ratio (AR) was then computed from the ratio of second area moments of *I* for the polygon [39]. Codes were implemented in MATLAB.


$$I=\left[\begin{array}{cc} \int x^{2}dA & \int xydA\\ \int xydA & \int y^{2}dA \end{array}\right],AR\bar{=}\sqrt{\frac{I_{1}}{I_{2}}}$$

### Statistical analysis

Statistical testing is done using a two-sample t-test. In figures, m represents the total number of time frames that were analyzed, and n represents total number of experimental replicates. Wherever n is not mentioned, it indicates one representative video and m represent total number of samples. The statistical tests were done using MATLAB.

## One-dimensional motor-clutch model of monolayer expansion

### Overview

As shown in (Fig 6a), the model is composed of cell monolayer, substrate and an intermediate layer of ligand which connects monolayer to substrate. Each of these layers is modelled as a collection of springs and dashpots (Kelvin-Voigt) connected in series. Connector springs between cell monolayer and ligand springs depict adhesion complexes represented by various proteins like Integrin, Paxillin, Vinculin and Talin which bind actin to extra-cellular matrix proteins [82] represented here by the ligand layer. The ligand layer is attached to the underlying substrate via links which represent the covalent attachment between collagen fibers and mod-PA gels in experiments. Additionally, the protrusive part of the monolayer is represented by protrusive nodes at each end of the cell layer which are acted upon by a constant force *F*_*p*_. Force is transmitted across cell-cell junctions and to the substrate via intermediate ligand layer from the monolayer edges [28]. Next, we summarize the different components of the model and its numerical implementation.

### Cell monolayer

Monolayer is modelled as a collection of springs connected in series. While protrusions are implemented only at the leading edge, cellular contractility is represented by the contraction force which gets exerted when cell springs get stretched after protrusion. This contraction force is transferred to the substrate mesh via the ligand mesh and represents the traction force exerted by the cell monolayer mesh.

### Ligand layer

We implemented aligned fibers in the 1-d by joining multiple ligand nodes in series to form connected ligands. Random fibers were modelled as disconnected ligands with every two nodes disconnected from the third node leading to discontinuity in ligand connectivity (Fig 6b and 6c). Every ligand node has equal binding probability to cell node which is set to 1. We vary this binding probability spatially as discussed in ‘*Haptotaxis*’ section later. Every ligand node is connected to substrate node via springs representing covalent linkages between collagen fiber and mod-PA gel [33].

### Substrate layer

Substrate is modelled as a series of springs connecting substrate nodes and have uniform stiffness. We vary the stiffness of substrate springs spatially while implementing ‘*Durotaxis*’ and discussed in later section.

### Model implementation

Force balance at every cell node ${x_{c}}_{i}$ reads (1), where *F*_*c-c*_ represents force at cell-cell junction (2) and *F*_*c-l*_ represents force at cell-ligand junction (3). *F*_*p*_ represents protrusive force and *F*_*c-l*_ only act on first node and last node (*i* = 1 *OR i = N*). Here, ${\varepsilon_{c}}_{i}\;$ denotes strain experienced by spring connecting nodes ${x_{c}}_{i}$ and ${x_{c}}_{i+1}$, and ${\dot{x_{c}}}_{i}$ denotes velocity of node ${x_{c}}_{i}$.


$${F_{p}+F}_{c-c}+F_{c-l}+k_{c}{\varepsilon_{c}}_{i}\;-k_{c}{\varepsilon_{c}}_{i+1}\;-\;\eta_{c}{\dot{x_{c}}}_{i}=0$$ (1)

$$F_{c-c}+k_{c}{\varepsilon_{c}}_{i-1}\;-k_{c}{\varepsilon_{c}}_{i}=0$$ (2)

$$F_{c-l}+k_{c-l}{\varepsilon_{c}}_{i}\;-k_{c-l}{\varepsilon_{l}}_{i}=0$$ (3)

Force balance at every ligand node ${x_{l}}_{i}$ reads (4), where *F*_*l−l*_ represents force at ligand-ligand spring junction (5) and *F*_*l−s*_ represents force at ligand-substrate spring junction (6).


$$F_{l-l}-F_{c-l}+F_{l-s}+k_{l}{\varepsilon_{l}}_{i}-k_{l}{\varepsilon_{l}}_{i+1}-\;\eta_{l}{\dot{x_{l}}}_{i}=0$$ (4)

$$F_{l-l}+k_{l}{\varepsilon_{l}}_{i-1}\;-k_{l}{\varepsilon_{l}}_{i}=0$$ (5)

$$F_{l-s}+k_{l-s}{\varepsilon_{c}}_{i}\;-k_{l-s}{\varepsilon_{l}}_{i}=0$$ (6)

Force balance at every substrate node ${x_{s}}_{i}$ reads (7), where *F*_*s−s*_ represents force at substrate-substrate spring junction (8).


$$F_{s-s}-F_{l-s}+k_{s}{\varepsilon_{s}}_{i}-k_{s}{\varepsilon_{s}}_{i+1}-\;\eta_{s}{\dot{x_{s}}}_{i}=0$$ (7)

$$F_{s-s}+k_{s}{\varepsilon_{s}}_{i-1}\;-k_{s}{\varepsilon_{s}}_{i}=0$$ (8)

For each incremental time step, the following algorithm is implemented:

1. Apply protrusive motor force to leader node only if it is at equilibrium (at rest).
2. Solve Eqs 1, 2 and 3 for nodes representing the cell mesh.
3. Solve Eqs 4, 5 and 6 for nodes representing the ligand mesh.
4. Solve Eqs 7 and 8 for nodes representing the substrate mesh.
5. The displaced cell leader node attaches to the nearest ligand node engaging clutch.
6. Check strain for every cell and perform cell division if strain is greater than the threshold division strain.

### Single contraction experiment

To check whether forces exerted by the contracting monolayer are different across connected and disconnect ligand conditions, we studied the cell mesh contraction after a single protrusion event. As shown in Fig 6d, we observe that cell mesh contracting on connected ligand comes to rest within 6 mins while for disconnected ligands, cell mesh comes to rest only at 21 mins (Fig 6e). Also, the cell mesh undergoes lesser displacement at the leading edge while contracting in case of connected condition. This is also shown by the traction plots (Fig 6f and 6g) where the substrate mesh on connected ligand condition undergoes lower contraction due to cell mesh getting stabilized quicker resulting in lesser traction forces compared to disconnect ligand condition. When we repeat protrusion event for connected condition, we see that cells can make three protrusions in the same time cells make one on disconnected ligand condition (Fig 6h and 6i) and for each protrusion, the resulting contractility driven tractions are lesser in magnitude to the traction for the single event taking place on disconnected ligand condition (Fig 6j and 6k). Mathematically, we can explain the above phenomenon as follows. In Fig 6b and 6c, when we consider force balance for ligands alone,

$$F_{cell-ligand}=\sum_{1}^{N}F_{ligand-substrate}$$ (9)

Clearly, *N* is greater for connected ligand condition (Fig 6b) when compared to disconnected ligand condition (Fig 6c). When we consider the fiber (red box) containing ligand (red spring), then *F*_*ligand-ligand*_ becomes an internal force and will cancel out. Only external forces *F*_*cell-ligand*_ and *F*_*ligand-subtrate*_ should play a part in the force balance. Also springs connecting ligands are much stiffer than springs connecting ligand to substrate or cell to ligand (*k*_*ligand-ligand*_ ≫ *k*_*ligand-substrate*_; *k*_*ligand-ligand*_ ≫ *k*_*cell-substrate*_). Thus, fiber is unlikely to deform due to external force *F*_*cell-ligand*_, meaning ligand springs undergo minimum compression/expansion.

When cells apply *F*_*cell-ligand*_ while contracting after a protrusion event, in case of connected ligand condition, this force is balanced by greater number of *F*_*ligand-subtrate*_. As a result, the substrate undergoes lesser deformation, and the contracting cell node comes to rest at a faster rate. *F*_*cell-ligand*_ remains the same across the two conditions since leader nodes experience same protrusion forces (*F*_*p*_).

### Parameter scan

Next, we let the simulation for both the conditions run for greater time steps. We then performed a scan of model parameters to check which parameter regimes preserve the above phenotype of faster migration and lower traction forces on connected ligands (Fig 7). We first vary the stiffness of cell springs (*k*_*c*_). In Fig 7a, we observe that for all the stiffnesses tested, cells on connected ligands travel faster yet apply lesser traction forces compared to disconnected ligand condition represented by the ratio of average leading-edge velocity to the average leading-edge traction forces. A higher ratio indicates higher migration efficiency. However, for each condition, we observe that increasing stiffness of the cell springs reduces migration efficiency. Since stiffness of cell springs is a measure of cell contractility, this plot shows that increasing contractility without increasing protrusive forces can negatively affect collective migration efficiency implying that there needs to be balance between protrusive and contractile forces for effective collective migration [61]. We next varied the cellular damping constant (*η*_*c*_) (Fig 7b), and observe that for all tested values, migration on connected ligand is more efficient than disconnected ligands, although increasing cellular damping reduces efficiency, implying that friction due to maturing cell-cell contacts leads to jammed amorphous motion [62]. Next, we varied substrate stiffness by varying the stiffnesses of individual springs (*k*_*s*_) comprising the substrate mesh (Fig 7c) and we observed that for all tested values, connected ligand condition displayed higher migration efficiency compared to disconnected condition. We also observe that increasing substrate stiffness increases migration efficiency [63]. Next, to measure the effect of ligand connectivity on migration efficiency in the disconnected condition, we varied the number of ligand nodes that were connected to each other. We observe that increasing ligand connectivity increases migration efficiency (Fig 7d). However, we observe that the monolayer’s expansion stalls after ~5 hours across all the tested parameters, a problem that is tackled next.

### Incorporating fluidization and spatial variation in cell stiffness and damping

Since the cell mesh begins to stall after ~5 hours of expansion, it indicates that the combined contractile forces within the mesh due to the expanded cell springs are dominating the protrusive forces at the leading edge, preventing the monolayer from further expansion. This also results in a convex curvature of the leading edge with progression of time arising from lower displacements upon protrusion because of increase contraction within (Fig 8a–8c). From experiments we have observed that cells undergo active fluidization within the monolayer to dissipate built-up stresses which in-turn allows the monolayer to further expand (Fig 4). Hence, we implement cell-division based fluidization within the monolayer, where cell springs divide when the strain reaches a threshold amount. We decided to incorporate a spatial variability in the ability of cells to divide following our experimental observations that cell divisions occur most frequently near the middle of monolayer rather than at the edges (Fig 4k and 4l). Thus, we implement a spatially varying threshold division strain (${\varepsilon_{c}}_{th}\left(x_{c}\right)$) according to Eq 10 (Fig 8j).


$${\varepsilon_{c}}_{th}\left(x_{c}\right)=\alpha_{\varepsilon }+\;\left(\gamma_{\varepsilon }\right)*\left(\delta_{\varepsilon }-\frac{1}{\sigma_{\varepsilon }\sqrt{2\pi }}e^{-\frac{1}{2}{\left(\frac{x_{c}-\mu_{\varepsilon }}{\sigma_{\varepsilon }}\right)}^{2}}\right)$$ (10)

Upon implementation of fluidization in our model, we see that cells are now able to able to continue expansion beyond the 5-hour mark (Fig 8d–8f). However, we still observe the convex shape of the leading edge with a brief period of stalling (between 8 and 13 hours) which discontinues once the forward propagating strain rate wave which originated at the center following cell division reaches the leading edge (Fig 8f). After which, the monolayer continues to protrude for the rest of the simulation duration (24 hours). Since the middle of the monolayer experiences maximum stretching and given that recent experiments have shown that leader cells have much higher stiffness compared to follower cells [64], we decided to implement a spatial variation in cell stiffness (*k*_*c*_(*x*_c_)) within the monolayer (Eq 11) with higher spring stiffness near the edges and softer springs at the center following distribution in Fig 8k.


$$k_{c}\left(x_{c}\right)=\gamma_{k}(\omega_{k}+\delta_{k}-\frac{1}{\sigma_{k}\sqrt{2\pi }}e^{-\frac{1}{2}{\left(\frac{x_{c}-\mu_{k}}{\sigma_{k}}\right)}^{2}})$$ (11)

Parallelly, we implemented a spatial variation in cellular damping constant (*η*_*c*_(*x*_*c*_)) (Eq 12), inverse with respect to cellular stiffness (*k*_*c*_(*x*_*c*_)) variation (Fig 8l), following the rationale that stiffer cells would have higher actin organization leading to more elastic characteristics (quicker deformations) compared to softer cells which would have delayed response to stress transfer, but more deformable hence viscous [65]. Additionally, since cell crowding due to proliferation occurs at the center, this leads to higher friction causing contact inhibition of motion compared to leading edges where there are more open spaces and lesser cellular densities. Thus, to capture 2-D contact inhibition in 1-D, the damping constant follows Eq 12.


$$\eta_{c}\left(x_{c}\right)=\beta_{\eta }+\gamma_{\eta }(\frac{1}{\sigma_{\eta }\sqrt{2\pi }}e^{-\frac{1}{2}{\left(\frac{x_{c}-\mu_{\eta }}{\sigma_{\eta }}\right)}^{2}}-\delta_{\eta })$$ (12)

As a result of these additions to the model (parameters in S2 Table), we see that the cells are now able to expand without stalling and the kymograph leading edge develops a concave morphology (Fig 8g–8i) like those seen in experiments (Fig 1e and 1f). We also see that including fluidization and spatial variations in cell stiffness (*k*_*c*_(*x*_*c*_)) and damping (*η*_*c*_(*x*_*c*_)) further improve the migration efficiency for both connected and disconnected condition but for each condition, connected ligands still have higher efficiency than disconnected condition (Fig 8m).

Next, using the above spatially varying cell stiffnesses, damping and threshold strain for division (S2 Table), we run simulations for both connected and disconnected conditions (S4 Fig). From the velocity kymographs (S4a and S4b Fig), we see that cells expand faster on connected fibers (also shown in leading-edge velocity comparison in plot c). Also, cells apply lesser forces on connected fibers as seen in traction kymographs (S4d and S4e Fig) and leading-edge traction comparison plot (S4f Fig) while developing lesser shear stresses within the cell layer (S4g and S4h Fig). However, cells on connected ligand condition travel twice as fast compared to disconnected condition, while applying tractions two times lesser on the same. We have seen in our experiments, that cells apply as much as two to three times lesser traction forces on AF (Fig 2o, 2p and 4m and 4n) while travelling 1.33–1.56 times faster on the same compared to RF (S2a and S3a Figs). Additionally, there are discrepancies in the strain-rate propagation between the simulated and experimental observations. We see that strain-rate propagation reaches the center on disconnected ligand (before 7-hour mark) faster than connected ligand (around 8-hour mark) (S4j and S4k Fig), which is contrary to what we observed in experiments (Fig 1i and 1j) and (Fig 1k and 1l). Additionally, we also observe that although χ_4_ peak (S4l Fig) is predicting unjammed motion for connected condition, it is not congruent with experimental χ_4_ peaks of AF and RF (Fig 1p). We next address these shortcomings in our model.

In plot Fig 2o, by comparing average traction exerted across the monolayer width (x-direction) between AF and RF we see that cells on AF apply steady forces from edge to center of monolayer; while on RF, high forces are only localized at the edges while monolayer interior exerts minimal forces. For cells to exert comparable tractions at the center and edge on AF indicates that focal adhesions are mature throughout the width of monolayer and capable of generating leading-edge level high tractions while on RF, mature focal adhesion exist only near the leading edges while in the interior of the monolayer only weak adhesions exist as indicated by the drastic tapering of traction forces. Since myosin proteins attached onto actin fibers cause contractile forces within monolayer [48,83], the spatial differences in traction distribution indicates differences in actin-myosin distribution between AF and RF. Thus, in addition to differences in ligand connectivity in our model, we next incorporate differences in actin-myosin contractility to further improve our model predictions.

### Implementing different spatial variations in stiffness, damping and fluidization across connected and disconnected ligand

Parallel to the differences noted in experimental leading-edge traction distribution on AF and RF (Fig 4m and 4n), we incorporate differences in actin-myosin contractility between connected and disconnected ligand condition in our in-silico model. Since in our model, stiffness of the cell spring (*k*_*c*_) is a measure of actin-myosin contractility, we decided to incorporate variability between the spatial variation of (*k*_*c*_ (*x*_*c*_)) in connected and disconnected condition by incorporating different spatial variation on cell spring stiffness (*k*_*c*_ (*x*_*c*_)) for connected and disconnected condition as shown in Fig 9a. We implemented inverse differences in spatial distribution of cell damping constant (*η*_*c*_ (*x*_*c*_)) (with respect to (*k*_*c*_ (*x*_*c*_)), following the assumption that uniform spatial cellular stiffness on connected ligand condition would imply uniform spatial cellular viscosity and vice-versa on disconnected ligand condition (Fig 9b). Additionally, we also incorporate differences in spatial variation of threshold division strain between connected and disconnected ligand condition (Fig 9c) given our observation of differences in position of maximum cell divisions between AF and RF (Fig 4k and 4l) and previous literature showing that cell stiffness modulates cell-division frequency [66,67]. Using these differences in parameters (enumerated in S3 Table), we run simulations (Fig 10).

### Implementing ‘*Haptotaxis*’

In our model, the differences between connected ligands and disconnected ligands occurs due to the differences in amount of ligand connectivity between the two conditions. This is shown in Fig 7 plot (d), where increasing the ligand connectivity leads to an increase in migration efficiency. However, in both our simulation conditions, the amount of ligand continuity is constant, i.e., for both the conditions, there are equal number of ligands for the leading-edge cell node to attach onto as it protrudes outward. We wanted to know if changing ligand continuity also has a similar effect on migration efficiency like ligand connectivity. *Haptotaxis* is a form of chemotaxis, where cells undergo directional migration upon exposure to gradients in ligand concentrations. In this phenomenon, emphasis is on changing the number of attachment-points that cells are exposed to as opposed to changing the interconnectivity of the attachment points, which we have studied so far. Thus, to test the effect of ligand continuity on migration efficiency, we implement *haptotaxis* using our model (Fig 10). To this end, we introduce a ligand binding probability (*ρ*_*l*_) in our model which limits the node binding capacity of the leading cell edge node. We thus mimic scarcity, or abundance of ligands by decreasing or increasing ligand binding probability. Spatial variation of ligand binding probability (*ρ*_*l*_(*x*_*c*_)) follows Eq 13. We further varied *α* to obtain multiple variations for *ρ*_*l*_(*x*_*c*_) (Fig 10k) and measured extent of *haptotaxis* for each condition (Fig 10l and 10m).


$$\rho_{l}\left(x_{c}\right)=({tan}^{-}(\alpha \left({-x}_{c}\right)+1.5)/3)$$ (13)

### Implementing *‘Durotaxis’*

In our model, the cell layer engages in crosstalk with the substrate layer via the ligand layer, as shown in Fig_7 plot (c), where increasing substrate stiffness (*k*_*s*_) improves migration efficiency. We next wanted to test whether our model can simulate *durotaxis*, a phenomenon where cells directionally migrate towards increasing substrate stiffness [28,83], to show broad applicability of our in-silico model (Fig 11). To this end, we incorporate a linear spatial gradient in substrate spring stiffness (*k*_*s*_(*x*_s_)) according to Eq 14. Further, we varied *k*_*min*_ to obtain multiple curves for *k*_*s*_(*x*_s_) (Fig 11k) and measured extent of *durotaxis* for each condition (Fig 11l).


$$k_{s}\left(x_{s}\right)=k_{min}+((k_{max}-k_{min})/x_{max})\left(x_{s}\right)$$ (14)

### Numerical implementation

Computational simulations were numerically implemented using the forward Euler method. All equations were implemented in a C++ code developed by the author. Multiple class definitions were borrowed from CHASTE library [84], an online code repository for cell-based simulations.

### Model limitations

We have implemented focal adhesions only at the leading edges in our model. As a result, we see that in our simulation results (Figs S4d, S4e, 9g and 9h), tractions are restricted to the leading edges for both connected and disconnected conditions. However, from our experiments, we clearly see that focal adhesions are getting engaged deep within the monolayer for AF while on RF, focal adhesions are restricted at the leading edges as indicated from the spatial evolution of *T*_*x*_ (across the monolayer width—x-direction) (Fig 2o). We decided to implement the focal adhesions only at the edges for both ligand arrangements because of the lack of experimental measurements on the binding and unbinding rates for inner focal adhesions across AF and RF. However, we hypothesize that the differential engagements of focal adhesions across monolayer depth on AF and RF leads to differential viscoelastic properties of the cells across both the conditions. Deeper engagement of focal adhesions across the depth of monolayer on AF would lead to uniform stiffness and viscosity across the depth while on RF, higher focal adhesions at the leading edges (reflected by higher tractions at the edges and tapering tractions from thereon) would indicate high stiffness at the monolayer edges, followed by tapering elasticity and increasing viscosity as we move towards the monolayer center. We have tried to capture these aspects (consequence of differential engagement of focal adhesions) by implementing varying viscoelasticity of cells across monolayer depth on connected and disconnected condition (Fig 9a–9c). We have roughly tried to keep the net elasticity in the monolayer for both connected and disconnected ligand condition same since cell spring elasticity in our model approximates the F-actin amount. Given that substrate stiffness is known to epigenetically regulate the nuclear architecture (by regulating relative euchromatin and heterochromatin compaction levels) thereby regulating gene expression and protein production (for example YAP) [85,86]. Since substrate stiffness across AF and RF is identical, we hypothesize that total amount of F-actin on AF and RF would be similar, however that amount gets redistributed differently dependent on the focal adhesion arrangement. Deeper focal adhesion engagement would mean longer (uniform) supracellular F-actin assembly (concentration) across the monolayer depth on AF while focal adhesion restricted to leading edges on RF would indicate shorter but more concentrated F-actin assembly at the leading edges.

## Supporting information

S1 Table References References for simulation parameters in S1 Table. (XLSX)

S1 Table Base set of parameters for simulations. (XLSX)

S2 TableParameters for spatial variation of ${\varepsilon_{c}}_{th}$, *k*_*c*_ and *η*_*c*_ (Eqs 10–12) resulting in plots in Fig 8j, 8k and 8l and simulations depicted in S4 Fig.(XLSX)

S3 TableParameters for spatial variation of ${\varepsilon_{c}}_{th}$, *k*_*c*_ and *η*_*c*_ (Eqs 10–12) resulting in plots in Fig 9a, 9b and 9c and simulations depicted in Fig 9d–9o.(XLSX)

S1 Fig (**a**) Modified soft polyacrylamide (mod-PA) gels are exposed to high strength magnetic field to orient collagen in particular direction. A PDMS stencil is deposited on the mod-PA gel. MCF-10A cells are seeded and allowed to attach only by the gap defined by PDMS stencil. When cells reach confluency, stencil is lifted, and cells start invading available space. Representative images of fluorescently labelled collagen-1 on AF (**b**) and RF (**c**). Scale bar is 50 μm. (**d** and **e**) Histogram depicting orientation angle distribution for collagen fibrils on AF (d) and RF (e). (**f**) FTIR plot showing presence of aldehyde group peak at 1722 cm^-1^. More than 400 fibers were analyzed for plots in **d** and **e**.
(TIF)

S2 Fig (**a**)Box-plot comparison of average velocity comparison for cells migrating on AF and RF (m = 157, n = 3, p = 1.98 x 10^−84^). (**b**) Average velocity of cells migrating on AF and RF as a function of time. Error is represented as standard error (m = 157, n = 3). (**c**) Time averaged cellular persistence over the entire duration of experiment (900 tracks for AF, 500 tracks for RF, p = 5 x 10^−18^). (**d**) Time average (m = 158) cellular density comparison for cells on AF and RF (p = 1.8 x 10^−127^). (**e**) Distribution of cellular aspect rations for cells on AF (red) and RF (blue). (**f**) Plot showing monolayer stress component *σ*_*xx*_ comparison between AF (red) and RF (blue) across the width of monolayer (x-direction) and averaged over the monolayer height (y-direction) (m = 157, n = 3).
(TIF)

S3 Fig(**a**)Box-plot comparison of average velocity comparison for cells migrating on AF and RF (m = 146, n = 2, p = 1.40 x 10^−28^). (**b**) Time-averaged spatial autocorrelation function of *v*_*x*_ for AF (red) and RF (blue), error is represented as standard error. (**c**) Strain energy imparted by monolayer on AF and RF averaged across entire duration of migration (m = 146, n = 2, p = 6.43 x 10^−222^). (**d**) Time averaged (m = 157) spatial correlation function of average-normal stresses in AF (red) and RF (blue), error is represented as standard error for n = 2 observations. (**e**-**g**) The alignment angle *φ* comparison between AF and RF at quintiles furthest (e), mid-distance (f) and closest (g) to the leading edge. In all three cases, distribution is narrower for AF indicating *plithotaxis* is more dominant in AF compared to RF. (**h**) Cumulative probability distribution $\bar{P}(\varphi )$ curves, from red to blue are at decreasing distance from leading edge for monolayer migrating on AF. (**i**) Cumulative probability distribution for monolayer migrating on RF.(TIF)

S4 Fig Simulated kymographs of (**a**,**b**) velocity for connected (left) and disconnected (right) conditions for ‘*fluidization with spatial variation in stiffness and damping’* condition. Plot comparing temporal evolution of leading-edge velocity between connected and disconnected ligands (**c**). Simulated kymographs of traction for connected (**d**) and disconnected (**e**) conditions. Plot comparing temporal evolution of leading-edge traction between connected and disconnected ligands (**f**). Simulated kymographs of shear stress for connected (**g**) and disconnected (**h**) conditions. Plot comparing average leading-edge velocity and average leading-edge traction velocity between connected and disconnected ligands (**i**). Simulated kymographs of strain rate for connected (**j**) and disconnected (**k**) conditions Plot comparing four-point susceptibility *χ*_4_ versus Δ*t* between connected and disconnected ligand conditions (**k**).
(TIF)

S5 Fig Plot showing changes in migration efficiency with changing ligand binding probability (*ρ*_*l*_). Low density corresponds to *ρ*_*l*_ = 0.7 and high density corresponds to *ρ*_*l*_ = 1.
(TIF)
