# Supplementary figures and images for: Fast yet force-effective mode of supracellular collective cell migration due to extracellular force transmission
Source: PLoS Comput Biol. 2025 Jan 9;21(1):e1012664. doi: 10.1371/journal.pcbi.1012664 (PMC11717197; doi:10.1371/journal.pcbi.1012664)

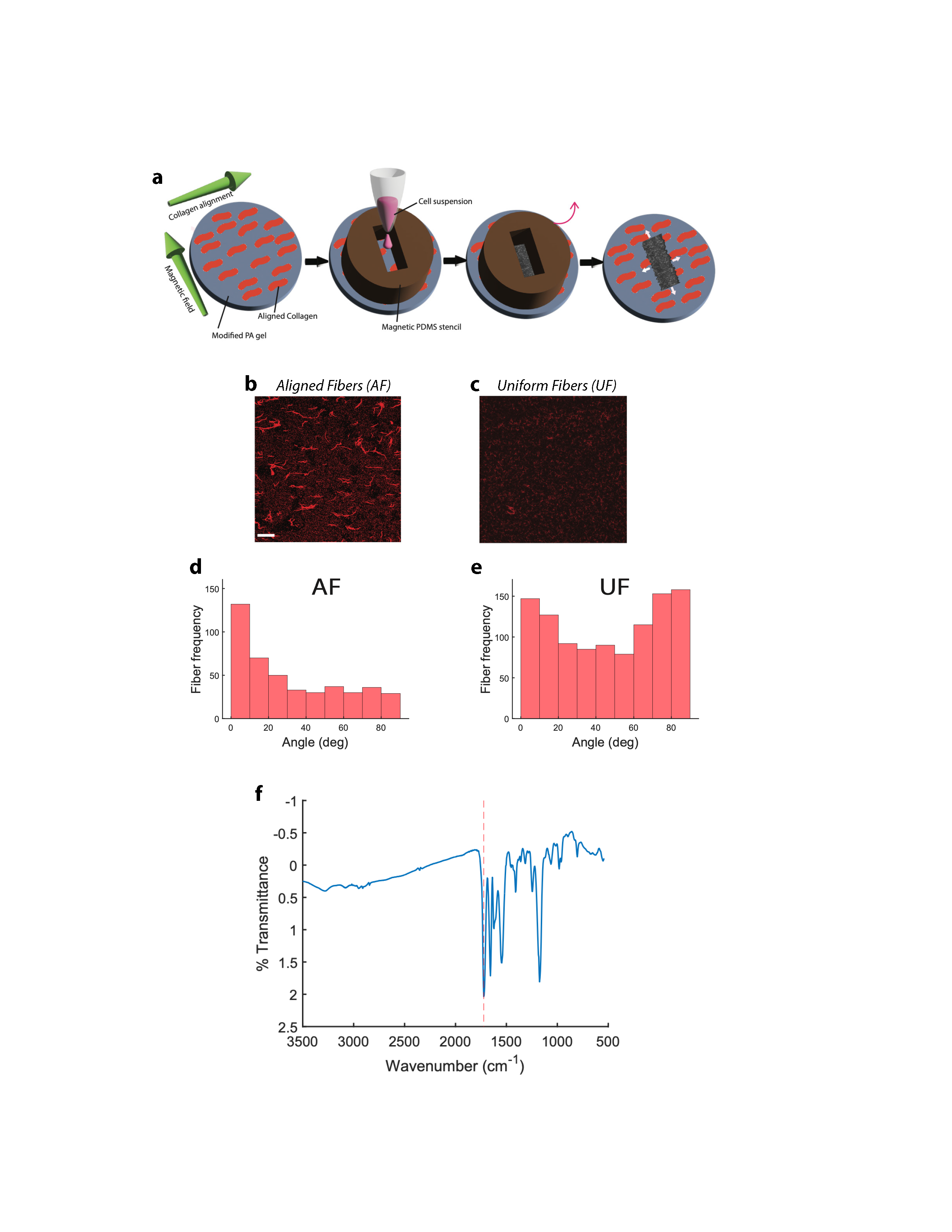

Supplement: S1 Fig — (a) Modified soft polyacrylamide (mod-PA) gels are exposed to high strength magnetic field to orient collagen in particular direction. A PDMS stencil is deposited on the mod-PA gel. MCF-10A cells are seeded and allowed to attach only by the gap defined by PDMS stencil. When cells reach confluency, stencil is lifted, and cells start invading available space. Representative images of fluorescently labelled collagen-1 on AF (b) and RF (c). Scale bar is 50 μm. (d and e) Histogram depicting orientation angle distribution for collagen fibrils on AF (d) and RF (e). (f) FTIR plot showing presence of aldehyde group peak at 1722 cm-1. More than 400 fibers were analyzed for plots in d and e. (TIF) [file pcbi.1012664.s005.tif]

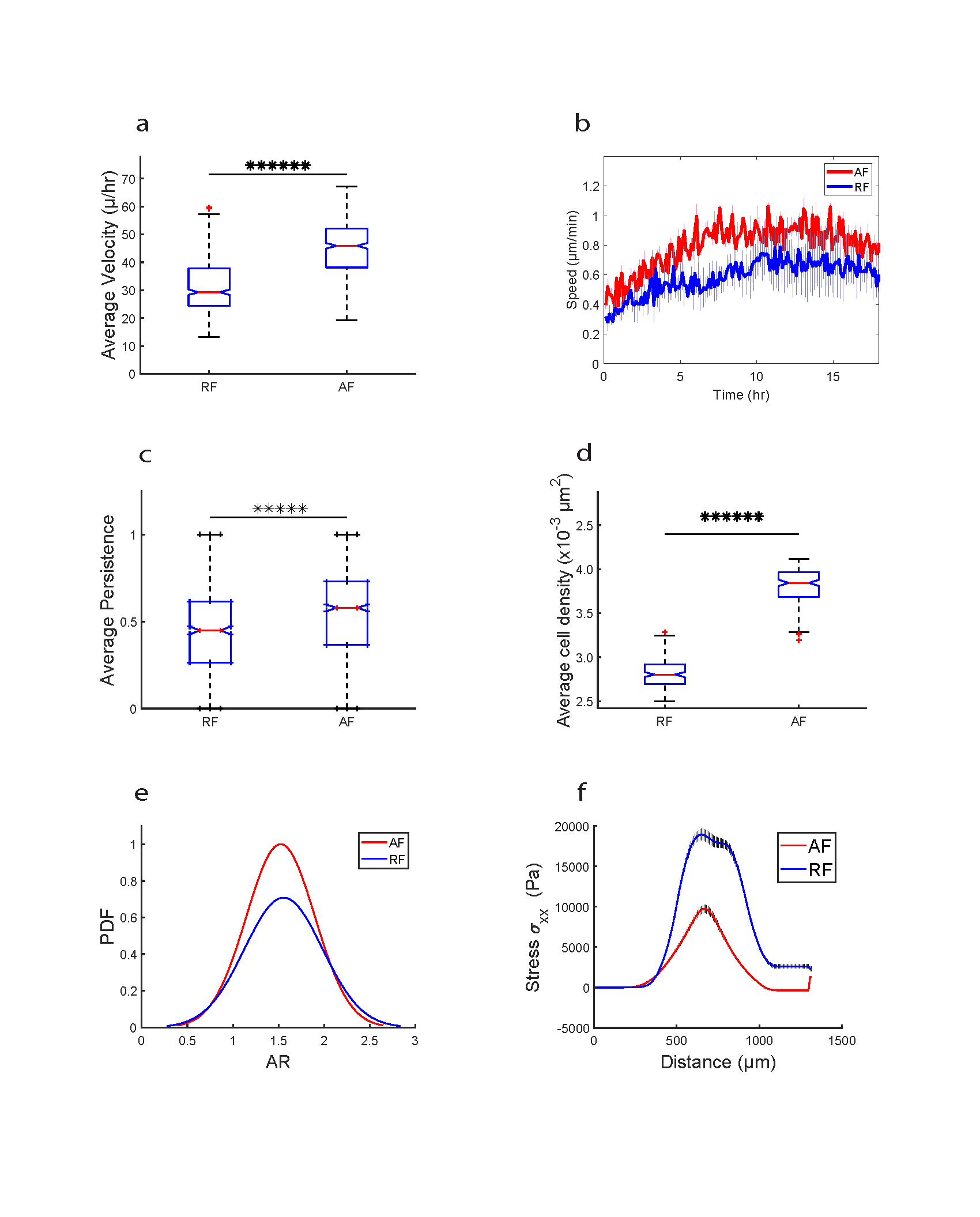

Supplement: S2 Fig — (a)Box-plot comparison of average velocity comparison for cells migrating on AF and RF (m = 157, n = 3, p = 1.98 x 10^−84^). (b) Average velocity of cells migrating on AF and RF as a function of time. Error is represented as standard error (m = 157, n = 3). (c) Time averaged cellular persistence over the entire duration of experiment (900 tracks for AF, 500 tracks for RF, p = 5 x 10^−18^). (d) Time average (m = 158) cellular density comparison for cells on AF and RF (p = 1.8 x 10−127). (e) Distribution of cellular aspect rations for cells on AF (red) and RF (blue). (f) Plot showing monolayer stress component σxx comparison between AF (red) and RF (blue) across the width of monolayer (x-direction) and averaged over the monolayer height (y-direction) (m = 157, n = 3). (TIF) [file pcbi.1012664.s006.tif]

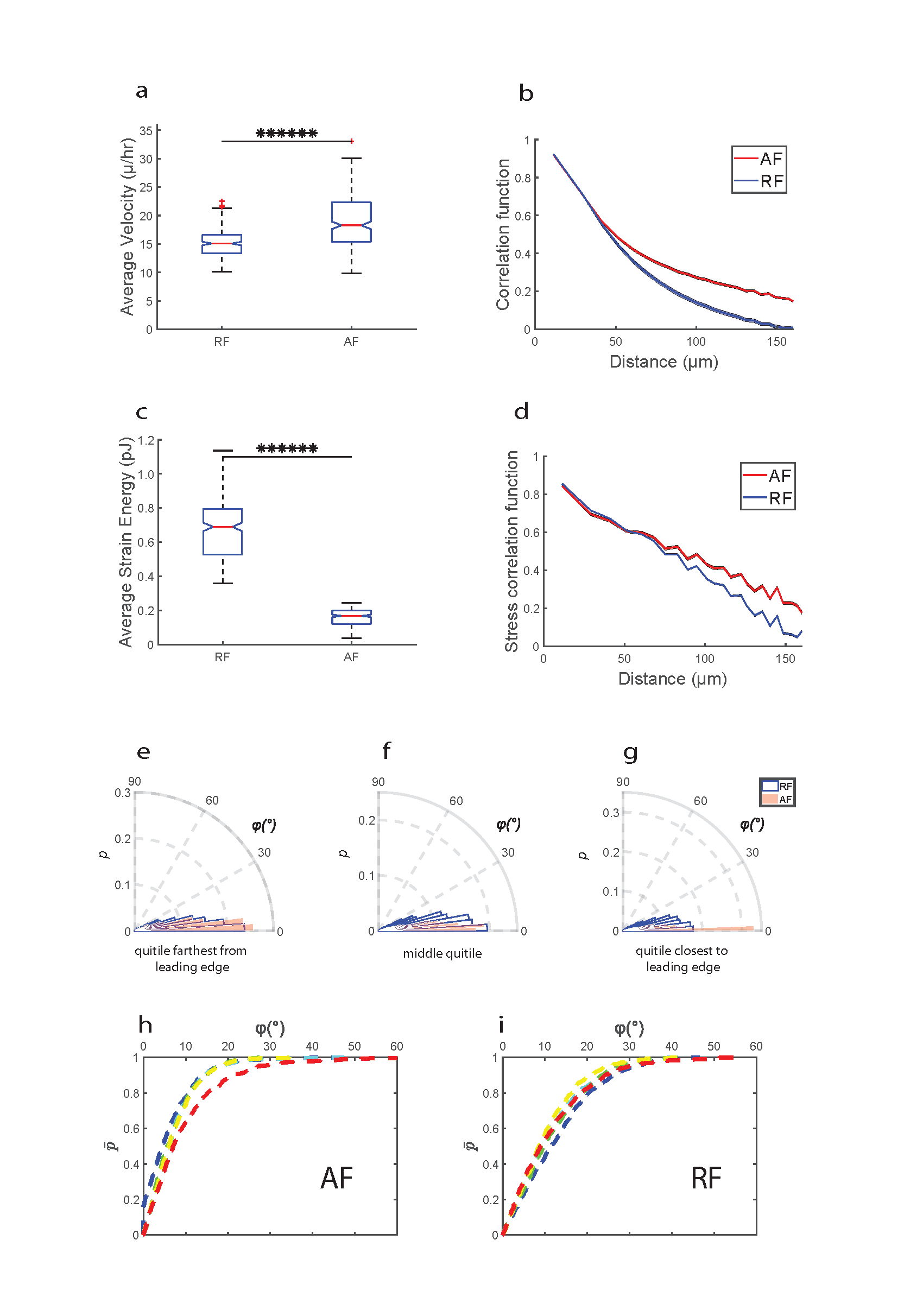

Supplement: S3 Fig — (a)Box-plot comparison of average velocity comparison for cells migrating on AF and RF (m = 146, n = 2, p = 1.40 x 10^−28^). (b) Time-averaged spatial autocorrelation function of vx for AF (red) and RF (blue), error is represented as standard error. (c) Strain energy imparted by monolayer on AF and RF averaged across entire duration of migration (m = 146, n = 2, p = 6.43 x 10−222). (d) Time averaged (m = 157) spatial correlation function of average-normal stresses in AF (red) and RF (blue), error is represented as standard error for n = 2 observations. (e-g) The alignment angle φ comparison between AF and RF at quintiles furthest (e), mid-distance (f) and closest (g) to the leading edge. In all three cases, distribution is narrower for AF indicating plithotaxis is more dominant in AF compared to RF. (h) Cumulative probability distribution P¯(φ) curves, from red to blue are at decreasing distance from leading edge for monolayer migrating on AF. (i) Cumulative probability distribution for monolayer migrating on RF. (TIF) [file pcbi.1012664.s007.tif]

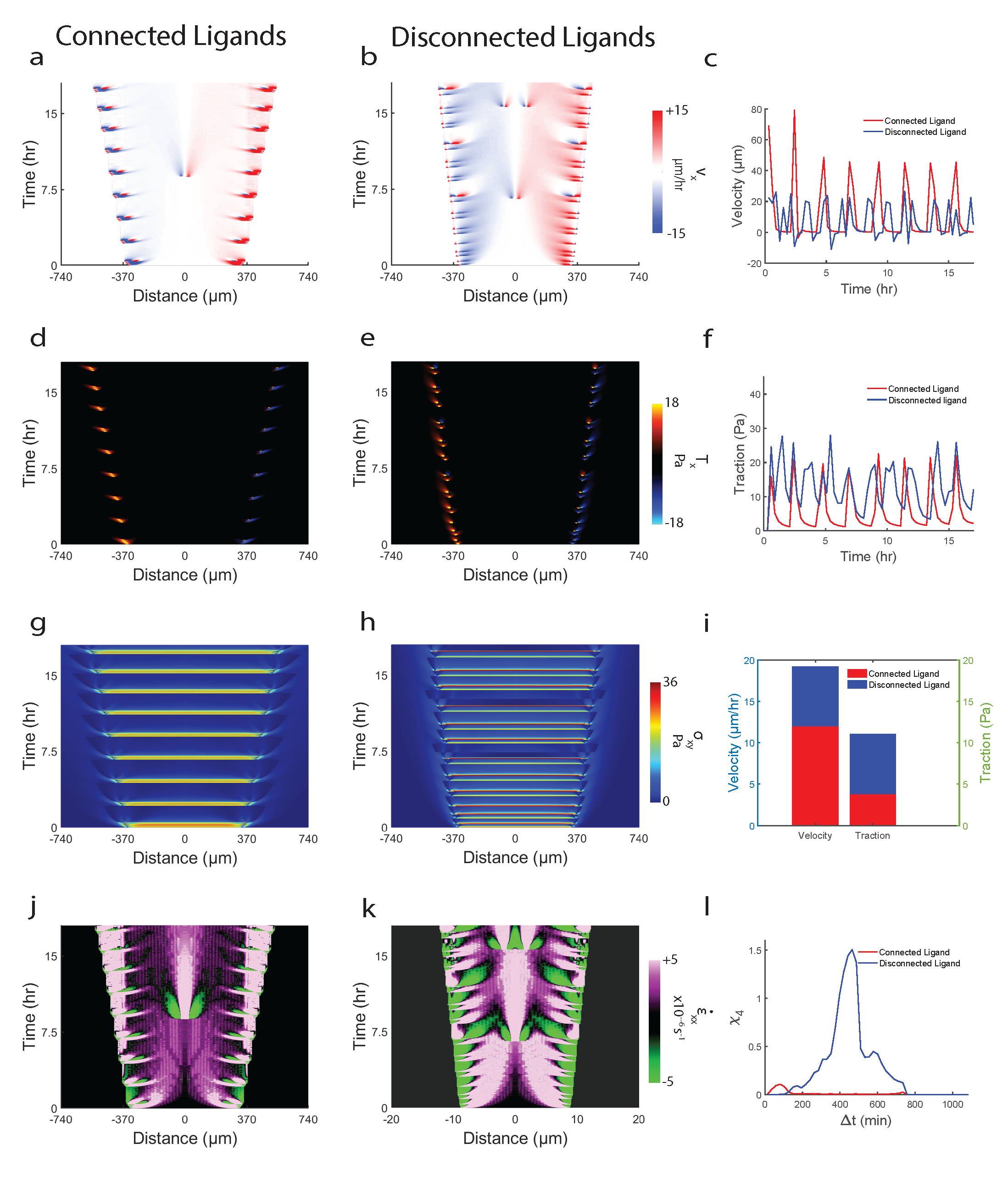

Supplement: S4 Fig — Simulated kymographs of (a,b) velocity for connected (left) and disconnected (right) conditions for ‘fluidization with spatial variation in stiffness and damping’ condition. Plot comparing temporal evolution of leading-edge velocity between connected and disconnected ligands (c). Simulated kymographs of traction for connected (d) and disconnected (e) conditions. Plot comparing temporal evolution of leading-edge traction between connected and disconnected ligands (f). Simulated kymographs of shear stress for connected (g) and disconnected (h) conditions. Plot comparing average leading-edge velocity and average leading-edge traction velocity between connected and disconnected ligands (i). Simulated kymographs of strain rate for connected (j) and disconnected (k) conditions Plot comparing four-point susceptibility χ4 versus Δt between connected and disconnected ligand conditions (k). (TIF) [file pcbi.1012664.s008.tif]

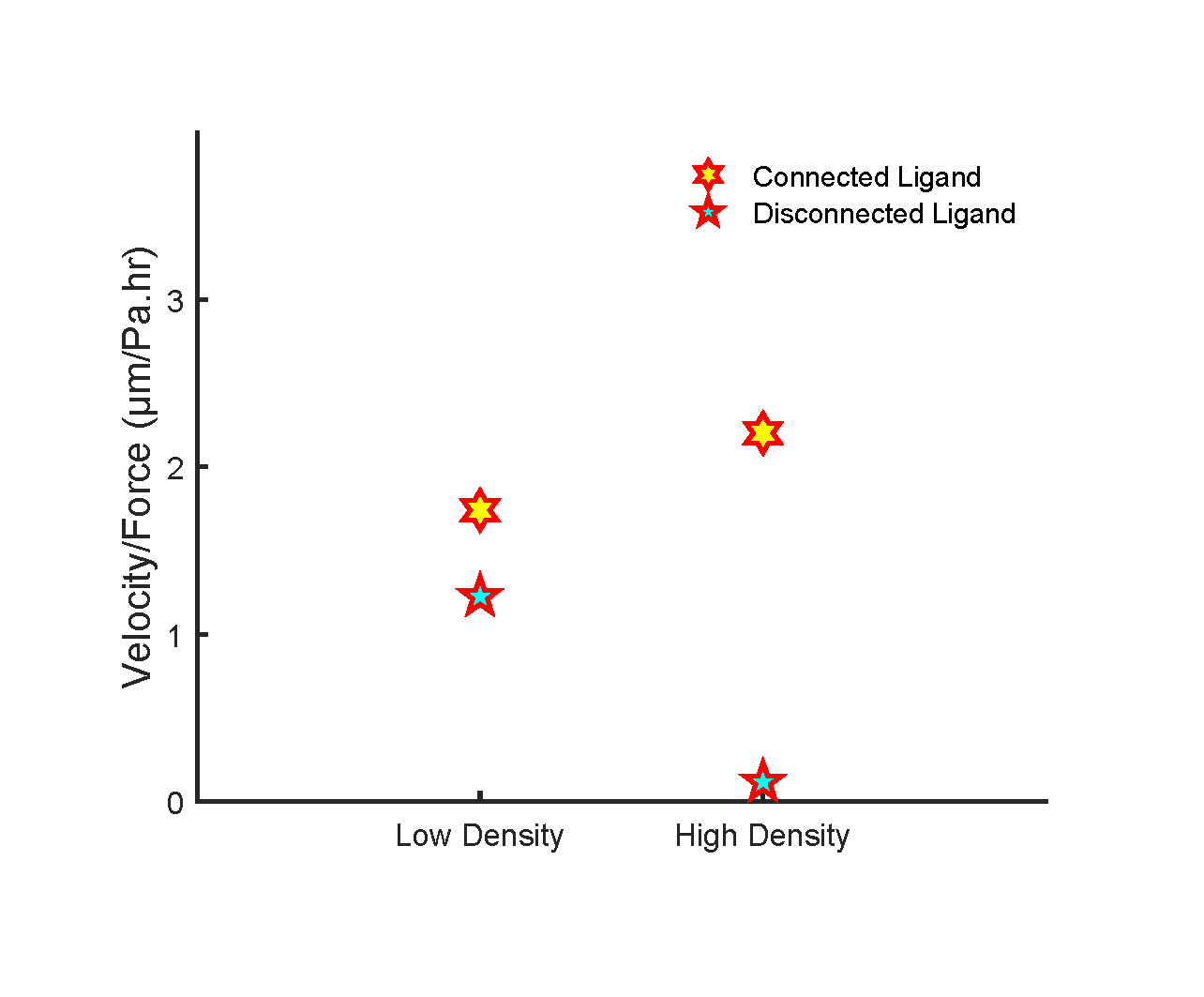

Supplement: S5 Fig — Plot showing changes in migration efficiency with changing ligand binding probability (ρl). Low density corresponds to ρl = 0.7 and high density corresponds to ρl = 1. (TIF) [file pcbi.1012664.s009.tif]
